# Bone-anchored prostheses for transfemoral amputation: a systematic review of outcomes, complications, patient experiences, and cost-effectiveness

**DOI:** 10.3389/fresc.2024.1336042

**Published:** 2024-04-02

**Authors:** Mayank Rehani, Tania Stafinski, Jeff Round, C. Allyson Jones, Jacqueline S. Hebert

**Affiliations:** ^1^ Division of Physical Medicine and Rehabilitation, Department of Medicine, Faculty of Medicine and Dentistry, College of Health Sciences, University of Alberta, Edmonton, AB, Canada; ^2^ Health Technology and Policy Unit, School of Public Health, College of Health Sciences, University of Alberta, Edmonton, AB, Canada; ^3^ Institute of Health Economics, Edmonton, AB, Canada; ^4^ Department of Physical Therapy, Faculty of Rehabilitation Medicine, College of Health Sciences, University of Alberta, Edmonton, AB, Canada; ^5^ Glenrose Rehabilitation Hospital, Edmonton, AB, Canada

**Keywords:** bone-anchored prosthesis, osseointegration, lower extremity, transfemoral, treatment outcome, postoperative complications, patient experience, cost-effectiveness analysis

## Abstract

**Introduction:**

Bone-anchored prostheses (BAP) are an advanced reconstructive surgical approach for individuals who had transfemoral amputation and are unable to use the conventional socket-suspension systems for their prostheses. Access to this technology has been limited in part due to the lag between the start of a new procedure and the availability of evidence that is required before making decisions about widespread provision. This systematic review presents as a single resource up-to-date information on aspects most relevant to decision makers, i.e., clinical efficacy, safety parameters, patient experiences, and health economic outcomes of this technology.

**Methods:**

A systematic search of the literature was conducted by an information specialist in PubMed, MEDLINE, Embase, CINAHL, Cochrane Library, the Core Collection of Web of Science, CADTH's Grey Matters, and Google Scholar up until May 31, 2023. Peer-reviewed original research articles on the outcomes of clinical effectiveness (health-related quality of life, mobility, and prosthesis usage), complications and adverse events, patient experiences, and health economic outcomes were included. The quality of the studies was assessed using the Oxford Centre for Evidence-Based Medicine Levels of Evidence and ROBINS-I, as appropriate.

**Results:**

Fifty studies met the inclusion criteria, of which 12 were excluded. Thirty-eight studies were finally included in this review, of which 21 reported on clinical outcomes and complications, 9 case series and 1 cohort study focused specifically on complications and adverse events, and 2 and 5 qualitative studies reported on patient experience and health economic assessments, respectively. The most common study design is a single-arm trial (pre-/post-intervention design) with varying lengths of follow-up.

**Discussion:**

The clinical efficacy of this technology is evident in selected populations. Overall, patients reported increased health-related quality of life, mobility, and prosthesis usage post-intervention. The most common complication is a superficial or soft-tissue infection, and more serious complications are rare. Patient-reported experiences have generally been positive. Evidence indicates that bone-anchored implants for prosthesis fixation are cost-effective for those individuals who face significant challenges in using socket-suspension systems, although they may offer no additional advantage to those who are functioning well with their socket-suspended prostheses.

## Introduction

Lower-limb amputation severely impacts physical function, psychological well-being, and social participation (1–8). Following a transfemoral (above-knee) amputation, the standard of care for restoring mobility is to fit the individual with a prosthesis that consists of a socket-suspension system to which the prosthetic components (such as the knee and foot) are attached. Approximately 86% of people with major lower-limb amputation are fit with a socket prosthesis (9). A trained prosthetist is required to custom-design the socket for each user according to the condition and shape of their residual limb. Suction to the residual limb or strapping around the pelvis is necessary for a socket to fit properly. Although prosthetic socket-suspension systems have evolved over the past few decades with substantial technological advancements, there are still limitations to their use. The socket must fit firmly to the residual limb to ensure comfort, transmit forces of the skeleton to the ground, and enable the movement of the residual limb to control the position of the prosthetic limb. The interface between the socket and the residual limb is one of the most crucial factors for the success of the prosthesis; however, discomfort and problems related to socket fit are common and have been shown to negatively affect the quality of life and mobility of the user (10–13). The problems that plague many prosthetic users are the lack of comfort, skin ulcers (14), inadequate or fluctuating suspension (15), tissue irritation, excessive heat and perspiration (14), poor control due to the motion of the soft tissue within the socket, and low confidence with mobility (12). Chronic skin problems and pain caused by friction between the residual limb and the prosthesis have been reported in 34%–63% of socket prosthesis users, reducing the use and function of the prosthetic device, quality of life, and body image satisfaction (12, 16–19). In addition, the socket can restrict the range of movement of the hip, leading to difficulties in sitting or participating in the activities of daily living. If the user experiences poor outcomes and problems with the socket, repeat visits to a physician or the prosthetist are required for assessment and adjustment. By some estimates, frequent refitting is typical in up to three-quarters of socket prosthesis users (11). Individuals who are unable to use the socket-suspension systems due to recurrent problems may completely abandon their prostheses (20, 21).

These problems spurred the development of new techniques to attach prosthetic components directly to a titanium implant that is inserted into the bone of the residual limb, obviating the need for a socket interface. Since titanium is naturally biocompatible (non-toxic and non-allergenic), titanium implant integrates with living bone tissue. This process, termed *osseointegration* (OI), results in a bone-anchored prosthesis in which the implant that extends percutaneously, i.e., through the skin, allows a direct functional and structural connection to the prosthetic components (22, 23). Bone-anchored implants have been used for dental and maxillofacial reconstructions for decades, and since the 1990s, they have been used for prosthetic reconstructions for individuals with transfemoral amputations (24). Bone-anchored prosthesis is a treatment option for various amputation levels in several areas worldwide.

### Types of implants for bone-anchored prostheses

Implants and protocols for bone-anchored prostheses have emerged and evolved over the past several years. Currently, there are two main types of fixations in use, namely, the screw-type (threaded) and press-fit type. Based on these two types of fixations, there are six types of implants for which evidence is available in the peer-reviewed literature (25). The first surgery for a person with transfemoral amputation occurred in 1990 in Sweden with the earliest design [called Osseointegrated Prostheses for the Rehabilitation of Amputees (OPRA)] (26). This is the only type of implant that relies on a screw-type fixation. As the name suggests, the titanium alloy implant is secured to the femur using a threading tool to cut spiral groove threads in the intramedullary cortex of the residual bone and then screwed into the femur. The OPRA technique is characterized by two surgical stages spaced 6 months apart. The success of the osseointegrated prostheses in Sweden spurred the design of implants in Germany in the late 1990s. This implant design diverted from screw-type fixation to intramedullary press-fit alloy devices similar to those used in joint arthroplasty. This led to the design of the Integral Leg Prosthesis (ILP; Orthodynamics, Germany), which was called the Endo-Exo Prosthesis (EEP; ESKA Orthopaedic, Germany) in its earlier iterations. In its latest iteration, the “third generation” of EEP is called Transcutaneous Osseointegrated Prosthetic Systems (TOPS) (27). The EEP and its successor ILP also rely on a two-stage surgical procedure, but the time between surgeries is reduced to 4–6 weeks. The Osseointegrated Prosthetic Limb (OPL; Permedica S.p.A., Italy) evolved from the experience with the ILP and is used in either a two- or single-stage surgery. More recently, the Bone Anchoring Device for Artificial Limbs (BADAL X) attachment using OTN Implants (the Netherlands) has been reported (28). Details on the varying designs and surgical and rehabilitation approaches are published elsewhere (25). Each type of implant has varying levels of evidence in the published literature on clinical efficacy outcomes and complications (25, 29, 30).

Two other types of implants are reported in the literature but are not the focus of this review since they are still in the development stage and/or lack adequate published literature for review. The Compress Device (Zimmer Biomet) was initially designed as a solution for large-gap limb salvage for patients with bone tumors, which is still used for that purpose (25). Since the Compress Device is a newer system, surgical techniques or rehabilitation guidelines have not yet been published. Despite having just finished its clinical trial, the Intraosseous Transcutaneous Amputation Prosthesis (ITAP; Stryker Orthopaedics; ClinicalTrials.gov no. NCT02491424) not be released due to a reported higher risk of infection and implant failure (31, 32).

Since the original surgeries in the 1990s in Sweden, various centers have begun providing BAP and publishing reports on clinical outcomes and complications, including centers in Germany, the Netherlands, Australia, the UK, the USA, and Canada. With the growing body of evidence on the outcomes of BAP from various groups around the world, there is increasing pressure on publicly and privately funded health insurance systems to make this procedure more widely available to all patients who could benefit. Implementation in new centers has been sporadic, typically facility determined, with discrepancies across private and publicly funded health systems. Inequitable access could be partly due to the unavailability of a single resource that brings together information on aspects most relevant for policymakers when making decisions about the provision of this new technology. Systematic reviews have covered outcomes (33–35), complications (36, 37), and implant design (25, 37); however, given the rapidly evolving evidence in this field, this review responds to the need for a single resource that presents an updated systematic review (by type of implant, where reported) of clinical efficacy outcomes, complications, patient preferences, and cost-effectiveness.

This review aims to present a systematic review to answer the four main questions that regulatory bodies and policymakers pose: What are the (a) clinical efficacy, (b) safety, (c) patient experience, and (d) cost-effectiveness of bone-anchored implants that enable attachment of prosthetic devices for persons with transfemoral amputations?

## Methods

We conducted a systematic review that adhered to the PRISMA 2020 checklists (Supplementary Appendices S1, S2) (38). An a priori protocol was drafted according to the PRISMA-*P* guidelines (39) and was made available online (40). An experienced medical information specialist developed and tested the search strategies through an iterative process in consultation with the review authors. Using the multifile and deduplication tool options in OVID, we searched Ovid MEDLINE ALL, including ePub Ahead of Print, In-Process & Other Non-indexed Citations, and Embase. In addition, we searched the Cochrane Library (Wiley), CINAHL (Ebsco), Web of Science Core Collection, and PubMed. All searches were performed on 14 March 2021 and updated on 31 May 2023. The strategies utilized a combination of controlled vocabulary (e.g., “bone-anchored prosthesis,” “osseointegration,” “bones of the lower extremity”) and keywords (e.g., “OPRA,” “osseo-anchor,” “femur”). Vocabulary and syntax were adjusted across the databases, and no language or date restrictions were imposed, although animal-only records were removed where possible. The results were downloaded and deduplicated using EndNote version 9.3.3 (Clarivate Analytics) and uploaded to Covidence (41). We performed a gray literature search using CADTH's Grey Matters and Google Scholar to ensure that no primary articles of interest were missed. The reference lists of the articles selected for full-text or included in this review were also searched for additional sources. Specific details regarding the strategies appear in Supplementary Appendix S3. Title and abstract screening and primary exclusion upon full-text review were carried out by two reviewers (MR and TS). Any conflicts at these stages were handled by consensus (between MR and TS), and a third reviewer (JSH) served as an arbiter when needed. Secondary exclusion upon full-text review was conducted by one reviewer (MR) and verified by another (JSH) who is a subject matter expert in prosthesis research.

### PICOTS elements

Population: Individuals with a unilateral or bilateral transfemoral amputation.
Intervention: Percutaneous osseointegrated/bone-anchored implants to which external prosthetic components are attached.
Comparator: Socket-suspension prosthesis systems or no prostheses.
Outcomes: (1) To assess clinical outcomes, health-related quality of life (HRQoL), and functional outcomes, such as mobility, and prosthesis usage, (2) clinical complications and adverse events, (3) patient experiences of benefits and challenges, and (4) any health economic variable.
Time: No restriction.
Studies: Articles published in peer-reviewed journals that were as follows: (1) experimental or observational studies with outcomes data on an intervention group and the comparator and studies with a pre-/post-design, (2) studies reporting specifically on complications and adverse events, (3) studies exploring patient experiences using qualitative methods, and (4) health economic evaluations based on, but not limited to, cost-comparison, cost–benefit analysis, cost-minimization analysis, cost-effectiveness analysis, or cost-utility analysis.

### Inclusion criteria

Primary peer-reviewed research reports meeting the PICOTS criteria were included.

### Primary exclusion criteria

Articles not meeting the PICOTS criteria were excluded at this stage. Other health technology assessments, literature reviews, case reports, opinion pieces, and editorials were also excluded.

### Secondary exclusion criteria

At this stage, articles that (1) reported on the same patients as those included in other studies, (2) did not report data for the transfemoral level separately, (3) self-identified as interim reports of longer-term follow-up studies, (4) described a bone-anchored prosthesis intervention not of interest, or (5) did not report data on the comparator group were also excluded.

### Data analysis and synthesis

Information was extracted by one reviewer (MR) from included studies only. Essential characteristics of the studies, e.g., implant type, city and country of the center publishing the study, funding source, study type, comparator, length of follow-up, details about external prosthetic components, number of participants, sex ratio, numbers of participants with unilateral or bilateral amputation, age of participants at treatment, time between amputation and surgery, etiology of the patients, and outcomes of interest, were extracted. Quantitative data on outcome measures of clinical efficacy were extracted, collated, and presented in tables.

### Quality and risk of bias assessment of included studies

To assess the quality of the literature, studies on clinical efficacy were reviewed by two reviewers (MR and TS) who assigned the Oxford Centre for Evidence-Based Medicine (OCEBM) Levels of Evidence (42) by consensus and carried out The Risk Of Bias In Non-randomized Studies of Interventions (ROBINS-I) (43). ROBINS-I is a tool designed to evaluate the risk of bias in the estimates of effectiveness or safety in studies that do not randomize the allocation of participants. It is well suited to cohort studies and single-arm trials as it assesses the risks to external validity due to confounding bias, selection bias, information bias, and reporting bias. The severity of these risks of bias is evaluated based on pre-established criteria and rated as “low,” “moderate,” “serious,” “critical,” and “no information,” where applicable.

The quality of literature focusing on complications was assessed by determining the OCEBM Levels of Evidence by one reviewer (MR) and verified by another (TS).

## Results

### Search results

**Figure 1:**
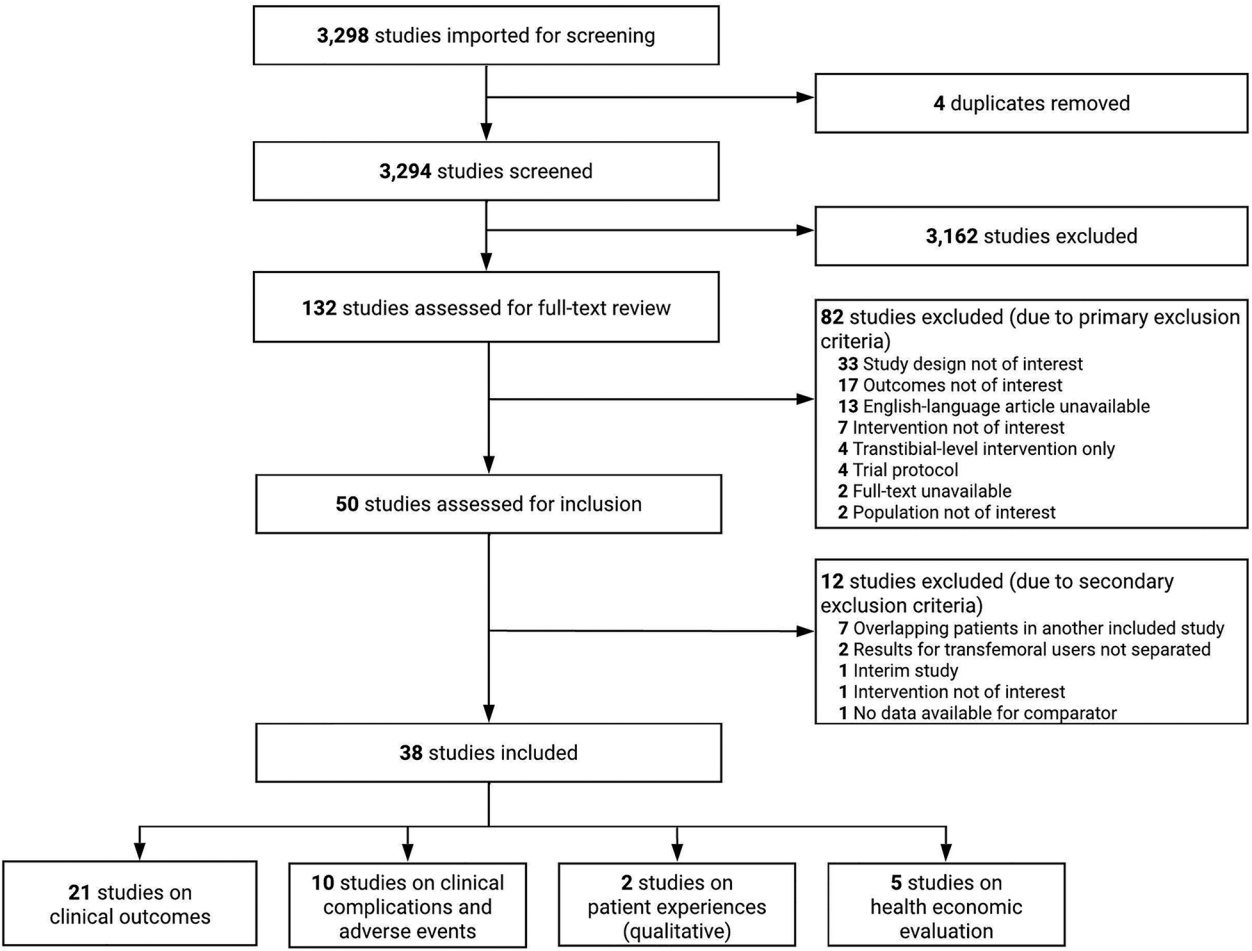
PRISMA flow diagram for the selection of studies for this review.

Figure 1 shows the study selection process as a PRISMA diagram. In total, 3,294 references were found in the 8 databases after removing the duplicates. After title and abstract screening by two reviewers, 132 articles were selected for full-text review. Eighty-two articles were excluded based on the primary exclusion criteria; however, an additional 12 were excluded based on the secondary exclusion criteria. Finally, 38 studies were included in this review. Twenty-one were on clinical efficacy outcomes (HRQoL, mobility, or prosthesis usage) with single-arm trial (pre-/post-intervention follow-up) design or cohort studies; nine case series (prospective or retrospective) and one cohort study were specifically reported on infectious or serious complications, two reported on patient experiences based on qualitative research methods, and five reported on health economic evaluations. Table 1 shows the list of studies (by implant type) included for evaluating clinical efficacy outcomes and information on study characteristics. Supplementary Table S1 shows the list of studies excluded at the secondary exclusion stage and the reasons for exclusion.

**Table 1:** Included studies on clinical outcomes.

Study	Implant type	OCEBM level of evidence	Country (city)	Funding source	Study type	Comparator	Length of follow-up reported	External prosthetic components (number of participants)	Number of patients (sex ratio) [unilateral:bilateral]	Mean age ± SD (if reported) at treatment (range or IQR) in years	Mean time ± SD (if reported) since amputation (range) in years	Etiology
Screw-type fixation												
Hagberg et al. (44)	OPRA	Level 2	Sweden (Mölndal)	Non-profit and commercial (Integrum AB)	Single-arm trial (pre-/post-comparison)	Socket (15/18) No prosthesis (3/18)	2 years	NR	18 (8 M: 10 F) [16:2]	45.44 (22–62)	15 (10m–33)	12 trauma, 5 tumor, 1 arterial embolus
Brånemark et al. (45)	OPRA	Level 2	Sweden (Mölndal)	Non-commercial^a^	Single-arm trial (pre-/post-comparison)	Socket (42/51) No prosthesis (9/51)	2 years	NR	At baseline: 51 (28 M: 23 F) [45:6] At follow-up: 48 (NR) [NR]	44 ± 12 (20–65)	12 (1–42)	33 trauma, 12 tumor, 6 other
Brånemark et al. (46)	OPRA	Level 2	Sweden (Mölndal)	Non-profit and government grants^a^	Single-arm trial (pre-/post- comparison)	Socket (42/51) No Prosthesis (9/51)	5 years	NR	At baseline: 51 (28 M: 23 F) [45:6] At follow-up: 40 (NR) [NR]	44 (20–65)	12 (1–42)	33 trauma, 12 tumor, 6 other
Matthews et al. (47)	OPRA	Level 2	United Kingdom (London)	No funding	Single-arm trial (pre-/post-comparison)	Socket	5 years	NR	18 (15 M: 3 F) [18:0]	34.77 (21–49)	NR	18 trauma
Zaid et al. (48)	OPRA	Level 2	USA (San Francisco)	NR	Single-arm trial (pre-/post-comparison)	Socket	1 year	NR	9 (7 M: 2 F) [NR]	45 (21–61)	12.9 (0–45)	4 trauma, 4 tumor, 1 infection
Hagberg et al. (49)	OPRA	Level 2	Sweden (Mölndal)	Non-profit and government grants^a^	Single-arm trial (pre-/post-comparison)	Socket	15 years	NR	At baseline: 111 (78 M: 33 F) [111:0] At follow-up: 11 (NR) [NR]	44.6 (17–70)	11.1 ± 10.8 (0–43)	75 trauma, 23 tumor, 3 emboli, 10 infection
Hagberg et al. (50)	OPRA	Level 2	Sweden (Mölndal)	Non-profit and government grants	Single-arm trial (pre-/post-comparison)	Socket (31/37) No Prosthesis (6/37)	10 years	NR (noted the lack of systematic documentation of external prosthetic device details as a limitation of the study)	At baseline: 51 (28 M: 23 F) [45:6] At follow-up: 37 (19 M: 18 F) [32:5]	44 (20–65)	12 ± 11 (1–42)	At baseline: 33 trauma, 12 tumor, 4 infection, 2 arterial embolus At follow-up: 25 trauma, 9 tumor, 1 infection, 2 arterial embolus
Press-fit type fixation												
Van de Meent et al. (51)	ILP	Level 2	The Netherlands (Nijmegen)	Non-commercial	Single-arm trial (pre-/post-comparison)	Socket	1 year	*Knee* Mechanical knee joint (3/22) Microprocessor-controlled knee; MPK (19/22) Did not report change in components post-intervention *Foot* NR	22 (18 M: 4 F) [21:1]	46.5 ± 10.7 (23–67)	16.4 ± 14.8 (2–45)	20 trauma, 2 tumor
Reetz et al. (52)	ILP	Level 2	The Netherlands (Nijmegen)	Non-profit and government grants	Single-arm trial (pre-/post-comparison)	Socket (38/39) No prosthesis (1/39)	5 years	NR	39 (30 M: 9 F) [38:1]	48.7 ± 13.9 (22–80)	12 (1–52)	29 trauma, 6 tumor, 3 infection, 1 other
Gailey et al. (53)	ILP	Level 3	United States (Miami, FL)	Government grant	Cohort study	Socket	N/A	*Knee* Mechanical knee joint (1/22) Group not specified MPK (21/22) *Foot* Dynamic response prosthetic foot (22/22)	22 OI group: 11 Socket group: 11 (Almost equal sex ratio reported in each group but numbers not reported for either group.)	Age (in years) at inclusion reported. OI group: 44.7 ± 14.9 Socket group: 49.6 ± 16.0	Mean time (in months) since amputation reported. OI group: 86.7 ± 102.3 Socket group: 149.7 ± 193.8 Mean time since OI for OI group not reported	Across both groups: 15 trauma, 3 tumor, 4 dysvascular disease
Al Muderis et al. (54)	ILP or OPL	Level 3	Australia (Sydney)	Commercial (Orthodynamic GmbH, Lübeck, Germany; Permedica S.p.A, Milan, Italy)^a^	Single-arm trial (pre-/post-comparison)	Socket (36/50) No prosthesis/wheelchair-bound (14/50)	Minimum: 1 year Mean: 21.5 months	NR (Authors mentioned that clinical outcomes may have been impacted due to fitting of superior external prosthetic components post-intervention)	50 (34 M: 16 F) [50:0]	48.4 (24–73)	NR	32 trauma, 8 tumor, 5 infection, 3 blast injury, 2 congenital
Leijendekkers et al. (55)	ILP or OPL	Level 2	The Netherlands (Nijmegen)	No funding^a^	Single-arm trial (pre-/post-comparison)	Socket (21/31) No prosthesis/wheelchair-bound (10/31)	1 year	Reported that all participants had the same prosthetic components post-intervention as they did pre-intervention (Details NR)	31 (17 M: 14 F) [29:2]	Median: 56 (IQR: 45–59)	Median: 6 (IQR: 3–26)	17 trauma, 7 tumor, 3 dysvascular disease, 4 other
Al Muderis et al. (56)	OPL	Level 3	Australia (Sydney)	Commercial (Permedica S.p.A, Milan, Italy; AQImplants GmbH, Ahrensburg, Germany; Osseointegration International Pty Ltd., Sydney, Australia)^a^	Single-arm trial (pre-/post-comparison)	Socket (12/22) No prosthesis/wheelchair-bound (10/22)	1 year	NR	22 (17 M: 5 F) [22:0]	46.2 (20–67)	NR	16 trauma, 4 tumor, 2 infection
McMenemy et al. (57)	OPL	Level 2	United Kingdom (Birmingham)	NR	Single-arm trial (pre-/post-comparison)	Socket	2 years	NR	7 (7M) [ 0: 7]	Median: 28 (IQR: 24–33)	Median: 96 months (IQR: 79–101)	7 trauma
Reif et al. (58)	OPL	Level 2	United States (New York, NY)	No funding	Single-arm trial (pre-/post-comparison)	Socket or none	6 months and 1 year	NR	18 (11 M: 7 F) [NR]	49.6 ± 12.0 (NR)	7.8 ± 8.8 (NR)	13 trauma, 3 infection, 2 vascular injury
Pospiech et al. (59)	EEP	Level 3	Germany (Lübeck)	No funding	Cohort study	Socket	N/A	*Knee* Mechanical knee joint (0/39) MPK (OI group: 22/22 Socket group: 17/17) *Foot* Triton (OI group: 16/22 Socket group: 12/17) C-Walk (OI group: 2/22 Socket group: 2/17) Kinterra (OI group: 2/22 Socket group: 1/17) Vari-Flex (OI group: 2/22 Socket group: 2/17) Reported no significant difference between groups based on type of prosthetic knees and feet	39 OI group: 22 (17 M: 5 F) [NR] Socket group: 17 (12 M: 5 F) [NR]	Age (in years) at inclusion reported. OI group: 48.7 ± 8.3 (NR) Socket group: 47.0 ± 12.3 (NR)	Mean time (in months) since amputation: OI group: 230 ± 138 (NR) Socket group: 241 ± 17 (NR) Mean time since OI for OI group: 66.8 ± 42.4 months (NR)	OI group: 15 trauma, 3 tumor, 4 other Socket group: 12 trauma, 4 tumor, 1 other
Örgel et al. (60)	EEP	Level 3	Germany (Hannover)	No funding	Cohort study	Socket	N/A	NR	69 OI group: 33 (17 M: 16 F) [NR] Socket group: 36 (18 M: 18 F) [NR]	Age (in years) at inclusion reported. OI group: 52.1 ± 9.7 (NR) Socket group: 48.6 ± 13.0 (NR)	NR Mean time since OI for OI group: 30.5 ± 41.5 months (NR)	OI group: 21 trauma, 3 tumor, 1 infection, 4 dysvascular disease, 4 other Socket group: 23 trauma, 3 tumor, 2 infection, 4 dysvascular disease, 4 other
Welke et al. (61)	EEP	Level 3	Germany (Hannover)	Government grant	Cohort study	Socket	N/A	*Knee* Mechanical knee joint (OI group: 0/20 Socket group: 0/17) MPK (OI group: 20/20 Socket group: 17/17) *Foot* NR	37 OI group: 20 (12 M: 8 F) [20: 0] Socket group: 17 (13 M: 4 F) [17:0]	Age (in years) at inclusion reported. OI group: 54.0 ± 8.2 (NR) Socket group: 62.0 ± 14.6 (NR)	OI group: 23.7 ± 13.2 (NR) Socket group: 25.2 ± 18.7 (NR) Mean time (in years) since surgery for the OI group: 6.6 ± 2.4 (NR)	OI group: 12 trauma, 4 tumor,1 infection, 2 dysvascular disease, 1 other Socket group: 9 trauma, 4 tumor, 0 infection, 3 dysvascular disease, 1 other
Atallah et al. (28)	OTN (reported as OFI-C for long femur and OFI-Y for short femur)	Level 2	The Netherlands (Nijmegen)	No funding^a^	Single-arm trial (pre-/post-comparison)	Socket	1 year	NR	69 (49 M: 20 F) [66:3]	OFI-C; *n* = 53 57 ± 14 OFI-Y; *n *= 16 50 ± 15	OFI-C; *n* = 53 6 (4–17) OFI-Y; *n* = 16 17 (8–28)	36 trauma, 8 tumor, 10 infection, 9 dysvascular disease, 3 congenital, 6 other^b^
Sinclair et al. (62)	POP	Level 2	United States (Salt Lake City, UT)	Government grant	Single-arm trial (pre-/post-comparison)	Socket	1 year	Reported that all participants had the same prosthetic components post-intervention as they did pre-intervention (Details NR)	10 (10 M: 0 F) [10:0]	48.8 ± 12.1 (32–68)	9.4 ± 5.7 (1–18)	5 blast injury, 2 motor vehicle collision, 3 other trauma
Davis-Wilson et al. (63)	Unspecified	Level 2	United States (Aurora, CO)	NR	Single-arm trial (pre-/post-comparison)	Socket	1 year	*Knee* Mechanical knee joint (0/9) MPK (9/9) *Foot* Dynamic carbon fiber (9/9) Reported no change to components post-intervention	9 (4 M: 5 F) [9:0]	47.67 ± 7.6 (38–58)	16.7 ± 12.4 (3–39)	NR trauma, NR tumor, 0 dysvascular disease, NR congenital limb deficiency

OPRA, Osseointegrated Prostheses for the Rehabilitation of Amputees; ILP, Integral Leg Prosthesis; OPL, Osseointegrated Prosthetic Limb; EEP, Endo-Exo Prostheses; POP, Percutaneous Osseointegrated Prosthesis.

NR, Not reported.

^a^ Some authors of this study have declared a potential financial conflict of interest in the company supplying the implant.

^b^ Etiology reported in this study combined data for 69 persons with transfemoral and 3 persons with through-knee amputation.

### Commonly reported outcomes

Commonly reported outcomes of clinical efficacy were HRQoL (as measured by patient-reported outcome measures such as SF-36, EQ-5D, and the Questionnaire for Persons with a Transfemoral Amputation; Q-TFA), mobility (as measured by a trained observer/clinician using instruments such as 2-min Walk Test; 2MWT, 6-min Walk Test; 6MWT, 10-min Walk Test; 10MWT, Timed-Up-and-Go Test; TUG), self-perceived mobility (as measured by PLUS-M), prosthesis usage, and safety parameters (which included the number and types of complications and adverse events). SF-36 was the most commonly reported generic HRQoL outcome measure. Q-TFA is a condition-specific HRQoL outcome measure to assess transfemoral prosthesis users’ quality of life and overall prosthetic situation (64). 2MWT, 6MWT, 10MWT, and TUG are performance-based tests administered and scored by a clinician. They measure the ambulatory potential of persons with lower-limb amputation with and without a prosthesis (65, 66).

### Types of studies and follow-up

**Figure 2:**
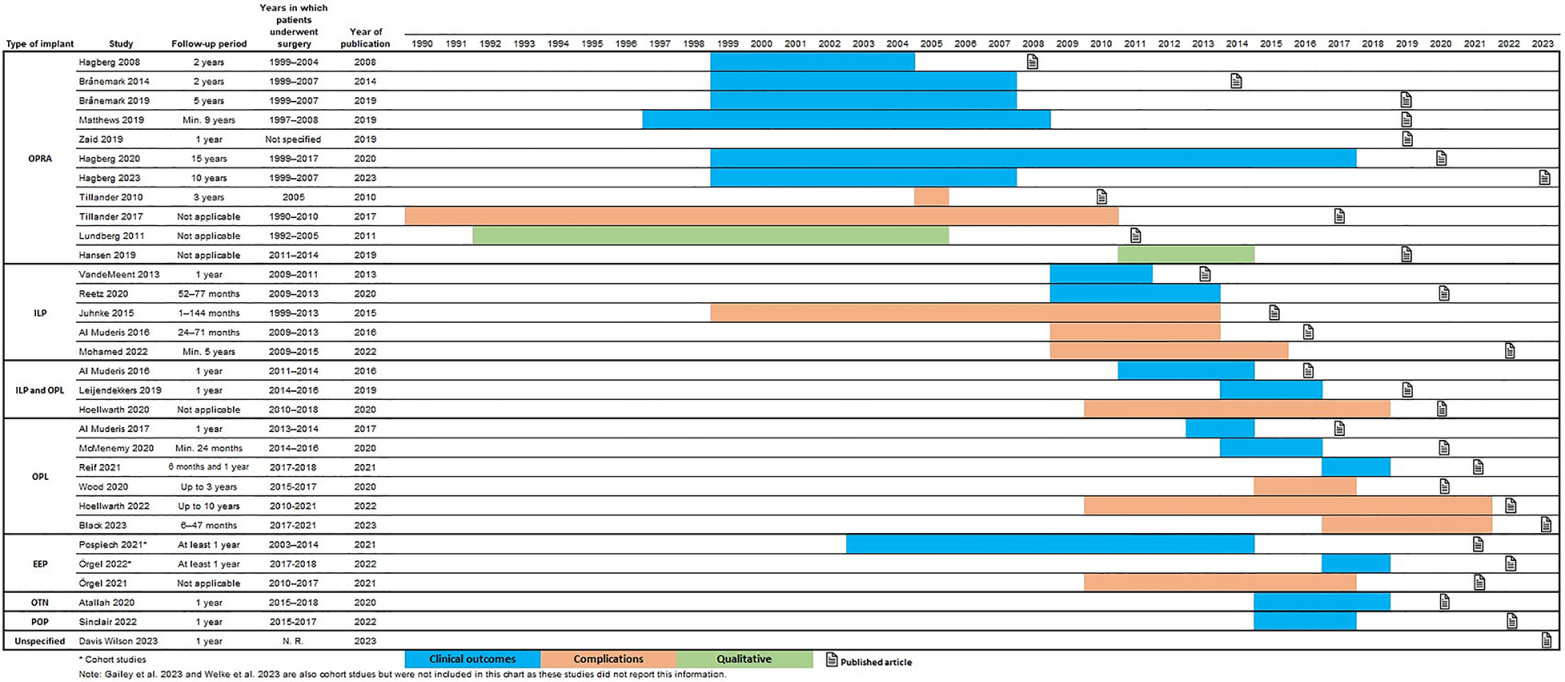
Lengths of follow-up of outcomes and complications and years of publication (by implant type).

Most of the included studies on clinical efficacy reported comparing outcomes before surgery (when patients used the comparator, i.e., socket-suspension systems or no prostheses) and after surgery with varying lengths of follow-up. Please refer to Figure 2 for a Gantt-chart-type depiction of the timeline of surgeries and follow-up in the included literature on the various bone-anchored implant types. Seventeen of the 21 studies were single-arm trials with pre-/post-intervention follow-up designs (where participant served as their own control) and 4 were cohort studies (where the comparisons were made between two distinct groups, the OI intervention group and the socket group). The shortest length of follow-up in the single-arm trials was 1 year postsurgery, and the longest was 15 years postsurgery. Twelve studies were either 1-year or 2-year postsurgery follow-ups. All seventeen single-arm trials of the 21 included studies on clinical efficacy outcomes also reported on complications in varying detail; the other four that did not were cohort studies. The two qualitative research studies were based on phenomenological methods. Two of the five health economic evaluations were based on cost-comparison analysis and three on cost-utility analyses.

### Quality of included studies and risk of bias

The quality of the included single-arm trials (pre-/post-intervention follow-up) and cohort studies was assessed using the OCEBM Levels of Evidence and ROBINS-I. The quality of the included case series was assessed using OCEBM Levels of Evidence. Supplementary Table S2 shows the quality assessment results for the studies on clinical outcomes. The general quality of evidence ranged from OCEBM Level 2 to 4, as most studies were single-arm trials or observational case series. The single-arm trials in this review were generally rated at Level 2 as they were deemed similar to well-designed clinical trials with several objective outcome measures and pre-/post-data on patients serving as their own controls. Cohort studies and case series (prospective or retrospective) were rated at Levels 3 and 4, respectively. Current practice patterns preclude study designs of higher methodological quality (such as RCTs) as difficulty with the socket-suspension system is generally considered a requirement for bone-anchored implants. Single-arm trials or cohort studies are therefore considered a methodologically robust and ethical way of comparing socket-suspension to bone-anchored implants.

The risk of bias (assessed using the ROBINS-I) was evaluated over four domains. The risk of confounding bias was generally considered moderate for most studies. In single-arm trials with pre-/post-design, the impact of any baseline confounding is typically minimal. In cohort studies, the risk of baseline confounding exists. One of the potential confounders identified in this review was the reporting of external prosthetic components that are attached to the bone-anchored implant. In studies where information about external prosthetic components was not reported, this risk was unknown and could have contributed to unmeasured confounding, therefore resulting in a moderate rating of confounding bias for several studies. The risk of selection bias was generally low. A lack of consistent reporting of how missing data was handled made the assessment of selection bias due to missing data challenging. Information bias due to the classification of interventions was considered low but was generally considered moderate due to the selection of outcome measures. The risk of reporting bias was generally rated as moderate but serious in two studies. The details on the choice of rating and rationale are presented in Supplementary Table S2.

### Patient selection criteria

The literature reported fairly consistent requirements for individuals to be selected for transfemoral OI surgery. Table 2 shows the inclusion and exclusion criteria in each of the included studies. The most common inclusion criteria are recurrent problems or the inability to use socket prostheses (28, 44–52, 54–58, 63), mature skeleton (28, 44, 45, 47–49, 52), or normal residual skeletal anatomy (44, 45, 47), the ability to comply with the treatment and follow-up requirements (45–48, 52, 54, 56, 62), and pre-surgical evaluation by a clinical team (44, 46, 51, 52, 57, 63) along with physical and medical examinations and imaging (44, 45, 47). There also are several contraindications to the OI surgery. The most common exclusion criteria are severe peripheral vascular disease (44–47, 49–52, 54, 56, 57), diabetes mellitus (28, 44–46, 49–52, 54, 56, 57, 62), treatment with chemotherapy (44–46, 49, 50, 52, 54, 56, 57, 63), exposure of the amputated limb to radiation (28, 52, 54, 56–58, 63), current treatment with corticosteroids (44–46) or immunosuppressive drugs (28, 54, 56, 57, 62), and pregnancy (44–46, 54, 56). The most common age range reported for patient selection in the included studies was between 18 and 70 years.

**Table 2:** Patient selection/inclusion and exclusion criteria in included studies on clinical outcomes*.*

Patient selection/inclusion criteria	Patient exclusion criteria
Transfemoral amputation (28, 44–52, 54–58, 62)	Severe peripheral vascular disease (44–47, 49–52, 54, 56, 57)
Patients with chronic pain or extremity dysfunction electing to undergo amputation with primary OI reconstruction (58)	Diabetes mellitus (44–46, 49–52, 54, 56, 57, 62) or severe diabetes (including medical history of multi-organ failure) (28)
Age below 70 years (44–47, 50)	Current treatment with chemotherapy (44–46, 49, 50, 52, 54, 56, 57, 63) or within 3 months of OI surgery (28)
Difficulty in using socket prosthesis (28, 44–52, 54–58, 63)	Exposure of amputated limb to radiation (52, 54, 56–58, 63) or within 3 months of OI surgery (28)
Previous or current use of a socket prosthesis (62)	Current treatment with corticosteroids (44–46) or immunosuppressive drugs (28, 54, 56, 57, 62)
Ability to comply with treatment and follow-up requirements (45–48, 52, 54, 56, 62)	Active infection (28, 48, 57, 62, 63) or within 6 months before the OI surgery (62)
Cause of primary amputation was congenital (55, 62, 63), trauma (55, 62, 63), tumor resection (55, 62, 63), or stable vascular disease (55)	Body weight more than 100 kg (44, 47, 48) or BMI ≥ 30 kg/m^2^ (62)
Mature skeleton (28, 44, 45, 47–49, 52)	Current pregnancy (44–46, 54, 56)
Normal residual skeletal anatomy (44, 45, 47)	Skin disease involving the amputated limb (45, 46)
“Sufficient” residual skeletal dimensions (49, 50)	Age less than 18 years (28, 54–56, 62, 63)
Assessment by a clinical team (orthopedic surgeon, physiotherapist, prosthetist) (44, 46, 51, 52, 57, 63)	Age less than 20 years (45, 46, 50)
Suitability for surgery assessed by medical and physical examinations and imaging (44, 45, 47)	Ongoing tobacco use (48, 54, 56, 57, 62)
Agreement to refrain from participation in high levels of physical activity (62)	Residual femoral length less than 9 cm (48)
Current or anticipated use of non-propulsive, passive microprocessor-regulated devices or passive non-microprocessor-regulated devices (62)	Residual femoral length less than 8 cm (51)
	Severely osteoporotic bone (58)
	Mental illness (52), psychological instability (56), disabling psychiatric disorder (54, 55, 58), or medical history of severe cognitive or psychiatric disorders (51)
	Bone deformity, dysplasia, metabolic disorders (28)
	Patients with opioid dependence not responsive to treatment (58)
	Demonstrated risk of substance abuse (62, 63)
	Non-traumatic etiology (63)
	Unstable heart condition (63)

### Clinical efficacy

Table 3 summarizes the results from the included studies on the clinical efficacy outcomes of interest (HRQoL, mobility, and prosthesis usage) for each type of implant. The participants in most included studies underwent OI surgery in their mid-40s, had unilateral transfemoral OI surgery, and underwent primary amputation due to trauma. Time between amputation and the OI surgery varied greatly between studies and ranged from 10 months (44) to 52 years (52). Three studies declared receiving funds, in whole or part, from commercial entities; whereas, 10 studies declared funding, in whole or part, from non-commercial and non-profit sources or government grants. Six declared having received no funding and three did not report having funding sources. The authors of seven studies declared a financial conflict of interest in the companies that supplied the implants. Seven studies were based on implants with screw-type fixation and 14 on those with press-fit fixation. All types of implants (screw-type or press-fit type) showed an improvement to varying degrees in HRQoL, mobility, and prosthesis usage when the pre-surgical condition (with socket prosthesis) of the patients is compared to their postsurgical condition. Most studies were based on the duration of follow-up of 1 year (28, 48, 51, 54–56, 58, 62, 63) or 2 years (44, 45, 57) post-intervention. There were three studies based on a 5-year follow-up (46, 47, 52) and one study each for 10-year (50) and 15-year (49) follow-ups, respectively, post-intervention.

**Table 3:** Reported clinical outcomes and results of quality of life, mobility outcomes, and prosthetic usage (by implant type).

Study	Implant type	Outcomes reported	Results (HRQoL)					Results (mobility)					Results (prosthetic usage)				Other			
Screw-type fixation																				
Hagberg et al. (44)	OPRA	Q-TFA, SF-36	Mean scores:					• Q-TFA prosthetic mobility score improved significantly, which indicates a reduction in reliance on walking aids and an improvement in walking ability and walking habits					• Q-TFA prosthetic use score improved significantly (*p* = 0.013) from a mean (SD) of 51.06 (41.52) at baseline to 82.89 (26.88) at follow-up • 17/18 using OI prosthesis with no restrictions at 2-year follow-up • 1/18 could not due to severe pain during weight bearing. This patient was reported to have implant loosening due to osteoporosis				NR			
			Q-TFA																	
			Subscore		Pre	Post	Sig													
			PUS		51.06	82.89	*p* = 0.013													
			Mobility		57.53	65.94	*p* = 0.001													
			Problem		38.07	16.53	*p* = 0.002													
			Global		37.73	72.12	*p* = 0.002													
			SF-36																	
			Subscore		Pre	Post	Sig													
			PCS		31	44	*p* = 0.001													
			MCS		55	50	n.s.													
			PF		31	60	*p* = 0.001													
			RP		38	68	*p* = 0.003													
			BP		53	72	*p* = 0.029													
			GH		75	79	n.s.													
			VT		61	62	n.s.													
			SF		80	83	n.s.													
			RE		78	73	n.s.													
			MH		76	77	n.s.													
			• Overall improvement was reported in condition-specific HRQoL (as measured by Q-TFA)																	
			• More generally, the improvement in SF-36 physical component score, physical functioning, physical role, and bodily pain subscales indicate an improved self-report of physical health																	
			• Slight decrease in SF-36 mental component score but not significant																	
Brånemark et al. (45)	OPRA	Q-TFA, SF-36, fixture cumulative survival rate	Mean scores:					• Mean Q-TFA prosthetic mobility score improved significantly					• Mean Q-TFA prosthetic use score improved • 47/51 patients using OI prosthesis at follow-up • 89% using prosthesis daily compared to 57% prior to OI				Cumulative survival rate at 2 years was 92%			
			Q-TFA																	
			Subscore		Pre	Post	Sig													
			PUS		47	79	*p* < 0.001													
			Mobility		52	70	*p* < 0.001													
			Problem		44	17	*p* < 0.001													
			Global		38	77	*p* < 0.001													
			SF-36																	
			Subscore		Pre	Post	Sig													
			PCS		74	76	*p* < 0.001													
			MCS		53	50	n.s.													
			PF		35	58	*p* < 0.001													
			RP		41	63	*p* < 0.001													
			BP		55	61	n.s.													
			GH		78	77	n.s.													
			VT		60	63	n.s.													
			SF		78	79	n.s.													
			RE		75	75	n.s.													
			MH		74	76	n.s.													
			• Q-TFA: Improved prosthetic use, mobility, global situation, and fewer problems • SF-36: Significant improvement in physical component scores and physical function and role physical subscales • Slight decrease in SF-36 mental component score but not significant																	
Brånemark et al. (46)	OPRA	Q-TFA, SF-36, fixture cumulative survival rate, revision-free rate	Mean scores:					• Q-TFA prosthetic mobility score improved significantly between baseline and at 5-year follow-up					• Q-TFA prosthetic use score improved significantly between baseline and at 5-year follow-up • At baseline 29/42 (69%) used their prostheses on a daily basis for at least 13 hours/day. At 5-year follow-up, 28/40 (70%) patients used their prosthesis for that long on a daily basis				• The 5-year fixture cumulative survival rate was 92% and the revision-free rate was 45%			
			Q-TFA																	
			Subscore		Pre	Post	Sig													
			PUS		47	86	*p* < 0.0001													
			Mobility		53	67	*p* < 0.0001													
			Problem		44	17	*p* < 0.0001													
			Global		38	74	*p* < 0.0001													
			SF-36																	
			Subscore		Pre	Post	Sig													
			PCS		32	41	*p* < 0.0001													
			MCS		53	51.1	n.s.													
			PF		35	60	*p* < 0.0001													
			RP		41	62	*p* < 0.0001													
			BP		55	61	n.s.													
			GH		78	82	n.s.													
			VT		60	64.3	n.s.													
			SF		78	83	n.s.													
			RE		75	78	n.s.													
			MH		74	77	n.s.													
			• At 5-year follow-up statistically significant improvements compared to baseline in all four Q-TFA scores and in the Physical Function, Role Physical, and Physical Component scores on the SF-36 • Improvements noted above were the same as (45) but no significant improvement between 2-year and 5-year follow-ups was reported																	
Matthews et al. (47)	OPRA	Q-TFA, SF-36	Mean scores:					• Q-TFA prosthetic mobility score improved significantly between baseline and 5-year follow-up • There were no significant differences between 2 years postsurgery and 5 years postimplantation					• Q-TFA prosthetic use score improved significantly between baseline and at 5-year follow-up • There were no significant differences between 2 years postsurgery and 5 years postimplantation				NR			
			Q-TFA																	
			Subscore		Pre	Post	Sig													
			PUS		NR	NR	*p* = 0.0001													
			Mobility		NR	NR	*p* = 0.0028													
			Problem		NR	NR	*p* = 0.0004													
			Global		NR	NR	*p* = 0.0001													
			SF-36																	
			Subscore		Pre	Post	Sig													
			PCS		NR	NR	*p* = 0.004													
			MCS		NR	NR	n.s.													
			PF		NR	NR	*p* = 0.0001													
			RP		NR	NR	n.s.													
			BP		NR	NR	n.s.													
			GH		NR	NR	n.s.													
			VT		NR	NR	n.s.													
			SF		NR	NR	n.s.													
			RE		NR	NR	n.s.													
			MH		NR	NR	n.s.													
			• HRQoL showed significant improvements up to 5 years after implantation • There were no significant differences between 2 years postsurgery and 5 years post-implantation • The physical scores, physical functioning, and physical component score in the SF-36 improved significantly from preoperative status and 2- and 5-year follow-ups • However, SF-36 scores did not change significantly between 2 and 5 years postoperatively • In the Q-TFA, there were significant improvements in all of the main scores between the preoperative period and 2- and 5-year post-implantation																	
Zaid et al. (48)	OPRA	Q-TFA, SF-36 subscales	Mean scores:					• Q-TFA prosthetic mobility score showed no significant change between baseline and 1-year follow-up					• Q-TFA prosthetic use score showed no significant change between baseline and 1-year follow-up				NR			
			Q-TFA																	
			Subscore		Pre	Post	Sig													
			PUS		32.9	62.8	n.s.													
			Mobility		52.3	65.4	n.s.													
			Problem		43.6	22.2	*p* = 0.02													
			Global		34.5	77.4	n.s.													
			SF-36																	
			Subscore		Pre	Post	Sig													
			PCS		NR	NR	N/A													
			MCS		NR	NR	N/A													
			PF		44	63.6	n.s.													
			RP		35	60	n.s.													
			BP		NR	NR	n.s.													
			GH		NR	NR	n.s.													
			VT		51.7	61.5	*p* = 0.009													
			SF		68.6	85.7	*p* = 0.02													
			RE		NR	NR	n.s.													
			MH		70	77	*p* = 0.002													
			• There was a significant improvement in the Q-TFA problem subscore • There was a trend toward improvement for all other subscores of the Q-TFA, although none reached significance • There were significant increases in three SF-36 health-related subscales (vitality, social functioning, and mental health)																	
Hagberg et al. (49)	OPRA	Q-TFA	Mean scores:					• The activity grade was higher at each time point as compared with the baseline. The activity grade (0–4) was assigned to each patient at each follow-up by the physiotherapist in the treating team, where 0 is no prosthetic activity and 4 is daily prosthesis use for full days without walking aids					NR				• The survival rate of the osseointegrated implant (the fixture) was 89% and 72% after 7 and 15 years, respectively • A total of 61 patients (55%) had mechanical complications			
			Q-TFA																	
			Subscore		Pre	Post	Sig													
			PUS		NR	NR	n.s.													
			Mobility		NR	NR	n.s.													
			Problem		NR	NR	*p* = 0.020													
			Global		NR	NR	*p* = 0.004													
			• Compared with before treatment, the patient-reported HRQoL was significantly better and remained so over the 15-year timeframe • Patients reported fewer problems and an improved overall situation at follow-up																	
Hagberg et al. (50)	OPRA	Q-TFA, SF-36	Mean scores:					• Q-TFA prosthetic mobility score improved significantly between baseline and at 10-year follow-up					• Q-TFA prosthetic use score improved significantly between baseline and at 10-year follow-up				NR			
			Q-TFA																	
			Subscore		Pre	Post	Sig													
			PUS		46.7	80.6	*p* < 0.001													
			Mobility		52.5	65.8	*p* < 0.001													
			Problem		43.9	16.3	*p* < 0.001													
			Global		37.7	74	*p* < 0.001													
			SF-36																	
			Subscore		Pre	Post	Sig													
			PCS		33	39	*p* = 0.001													
			MCS		53	52	n.s.													
			PF		35	58	*p* < 0.001													
			RP		41	53	n.s.													
			BP		55	57	n.s.													
			GH		78	74	n.s.													
			VT		74	80	n.s.													
			SF		75	74	n.s.													
			RE		78	80	n.s.													
			MH		60	65	n.s.													
			•At 10-year follow-up, statistically significant improvements in all four Q-TFA scores and in the Physical Functioning and the Physical Component scores on the SF-36																	
Press-fit type fixation																				
Van de Meent et al. (51)	ILP	Q-TFA global score, prosthesis use in hours, 6MWT, TUG, prosthesis use (in hours)	Mean score:					Mean scores:					Mean score:				• Q-TFA global score (68% higher) and prosthesis use (45% higher) significantly improved with OI prosthesis compared to socket prosthesis			
			Q-TFA					6MWT (in meters)					Prosthesis use (in hours/week)							
			Subscore		Pre	Post	Sig			Pre	Post	Sig		Pre	Post	Sig				
			Global		39	63	*p* = 0.001	Distance		321	423	*p* = 0.002	Hours/week	56	101	*p* < 0.001				
			•The Q-TFA mean global score improved significantly from 39 to 63					TUG (in seconds)					• Prosthesis use improved by 45% and increased to 101 hours/week with OI compared to 56 hours/week with socket							
										Pre	Post	Sig								
								Time		15.1	8.1	*p* = 0.002								
								• Significant improvements in 6MWT (27% increase) and TUG (44% faster)												
Reetz et al. (52)	ILP	Q-TFA prosthetic use score and global score	Median scores:					NR					• The Q-TFA median prosthetic use score improved significantly from 71 to 100				NR			
			Q-TFA																	
			Subscore		Pre	Post	Sig													
			PUS		71	100	*p* < 0.001													
			Global		33	75	*P* < 0.001													
			• The Q-TFA median global score improved significantly from 33 to 75 • Bone-anchored press-fit prostheses lead to greater prosthesis use and overall health-related quality of life even up to 5-years postsurgery follow-up																	
Gailey et al. (53)	ILP	10 MWT^a^, TUG, Activities-specific Balance Confidence (ABC) Scale, PLUS-M	NR					Mean scores:					NR				• No significant differences found between groups in Activities-specific Balance Confidence (ABC) Scale			
								10MWT (in meters/second)												
										OI group	Socket group	Sig								
								Speed		0.81	0.96	n.s.								
								TUG (in seconds)												
										OI group	Socket group	Sig								
								SSWS		12.39	11.27	n.s.								
								FWS		9.72	9.5	n.s.								
								PLUS-M												
										OI group	Socket group	Sig								
								T-score		59.17	53.50	n.s.								
								• No significant differences found between groups in TUG (at self-selected walking speed; SSWS, or fastest walking speed; FWS), or PLUS-M												
Al Muderis et al. (54) (Numbers of patients receiving ILP or OPL not specified)	ILP or OPL	Q-TFA, SF-36, AMPRO, 6MWT, TUG	Mean scores:					Mean scores:					NR				NR			
			Q-TFA					6MWT (in meters)												
			Subscore		Pre	Post	Sig			Pre	Post	Sig								
			PUS		NR	NR	N/A	Distance		281	419	*p* < 0.001								
			Mobility		NR	NR	N/A	TUG (in seconds)												
			Problem		NR	NR	N/A			Pre	Post	Sig								
			Global		47.82	83.52	*p* < 0.001	Time		14.59	8.74	*p* < 0.01								
			SF-36					• All 14 participants who were wheelchair bound preoperatively could not perform the TUG and 6MWT were able to do so postoperatively • Their postoperative scores were comparable with those of the patients who were walking preoperatively												
			Subscore		Pre	Post	Sig													
			PCS		37.09	47.29	*p* < 0.001													
			MCS		NR	NR	N/A													
			PF		NR	NR	N/A													
			RP		NR	NR	N/A													
			BP		NR	NR	N/A													
			GH		NR	NR	N/A													
			VT		NR	NR	N/A													
			SF		NR	NR	N/A													
			RE		NR	NR	N/A													
			MH		NR	NR	N/A													
			• Significant improvement in SF-36 physical component summary and Q-TFA global score																	
Leijendekkers et al. (55) (17 ILP, 15 OPL)	ILP or OPL	Q-TFA prosthetic use, Q-TFA global, 6MWT, TUG, prosthetic comfort score	Median score:					Mean scores:					• Q-TFA prosthetic use score increased at 6- and 12-month follow-ups compared to baseline				• Mean prosthetic comfort score (1–10) increased significantly (*p* < 0.001) from 5.4 to 8.2 between baseline and 12 months			
			Q-TFA					6MWT (in meters)												
			Subscore	Pre	6m Post	12m Post	Sig		Pre	6m Post	12m Post	Sig								
			PUS	52	90	100	N/A	Distance	319	284	313	*p* = 0.038								
			Mean score:					TUG (in seconds)												
			Q-TFA						Pre	6m Post	12m Post	Sig								
			Subscore	Pre	6m Post	12m Post	Sig	Time	13.0	12.8	11.3	*p* = 0.005								
			Global	48	69	70	*p* < 0.001	• TUG showed no change at 6-month follow-up but improved significantly at 12-month follow-up compared to baseline • Out of the 31 transfemoral, 10 were wheelchair-bound before OI surgery, at 6 months and 12 months postsurgery 0 patients were wheelchair-bound												
			• HRQoL (measured by Q-TFA global score) increased significantly at 6- and 12-month follow-up compared to baseline																	
Al Muderis et al. (56)	OPL	Q-TFA,SF-36, 6MWT, TUG	Mean scores:					Mean scores:					NR				N.R.			
			Q-TFA					6MWT (in meters)												
			Subscore		Pre	Post	Sig			Pre	Post	Sig								
			Global		NR	NR	*p* < 0.05	Distance		NR	NR	*p* < 0.05								
			SF-36					TUG (in seconds)												
			Subscore		Pre	Post	Sig			Pre	Post	Sig								
			PCS		NR	NR	*p* < 0.05	Time		NR	NR	*p* < 0.05								
			MCS		NR	NR	N/A	• 6MWT showed significant improvement (128%) at follow-up over preoperatively • TUG showed a significant reduction (30%) at follow-up than preoperatively												
			PF		NR	NR	N/A													
			RP		NR	NR	N/A													
			BP		NR	NR	N/A													
			GH		NR	NR	N/A													
			VT		NR	NR	N/A													
			SF		NR	NR	N/A													
			RE		NR	NR	N/A													
			MH		NR	NR	N/A													
			• Q-TFA global and SF-36 physical component scores were significantly higher at follow-up than preoperatively																	
McMenemy et al. (57)	OPL	SF-36, 6MWT, TUG	Mean scores:					Mean score:					NR				NR			
			SF-36					6MWT (in meters)												
			Subscore		Pre	Post	Sig			Pre	Post	Sig								
			PCS		34.65	54.5	*p* = 0.018	Distance		248	402	*p* = 0.018								
			MCS		41.55	58.19	*p* = 0.018	Median score:												
			• The physical component score (PCS) and mental component score (MCS) of the SF-36 demonstrated a statistically significant improvement representing a change from “below” or “well below” to “same or above” level expected of an age- and gender-matched able-bodied population					TUG (in seconds)												
										Pre	Post	Sig								
								Time		N/A	10.6	N/A								
								• The distance covered in the 6MWT improved by 154 m from baseline to 24-month follow-up • Preoperatively, no patient was able to perform the TUG test reliably and safely due to stump and balance problems. At the last postoperative review, all patients were able to perform the test with a median time of 10.6 s												
Reif et al. (58) (*Data on individuals with transfemoral amputation provided by the corresponding author*)	OPL	Q-TFA, PROMIS (Function, Pain Intensity, Pain Interference), LD-SRS^b^, 2MWT^c^, 6MWT, and EQ-5D	Mean scores:					Mean scores:					• Q-TFA prosthetic use score increased at follow-up compared to baseline				PROMIS			
			Q-TFA					2MWT (in meters)									Scale	Pre	Post	Sig
			Subscore		Pre	Post	Sig			Pre	Post	Sig					Function	34.00	40.53	Yes (*p*-value NR)
			PUS		49.77	75.23	Yes (*p*-value NR)	Distance		195.4	336.4	Yes (*p*-value NR)					Pain Intensity	46.60	44.39	Yes (*p*-value NR)
			Mobility		45.55	66.29	Yes (*p*-value NR)	6MWT (in meters)									Pain Interference	57.10	52.39	Yes (*p*-value NR)
			Problem		51.00	20.17	Yes (*p*-value NR)			Pre	Post	Sig								
			Global		17.60	77.08	Yes (*p*-value NR)	Distance		564.6	955.0	Yes (*p*-value NR)								
			EQ-5D					• 2MWT and 6MWT scores improved significantly at follow-up compared to baseline												
					Pre	Post	Sig													
			Index		0.62	0.66	n.s.													
			• Q-TFA prosthetic use score, mobility score, and global score were significantly improved at follow-up compared to baseline • Q-TFA Problem score was significantly reduced at follow-up compared to baseline. • EQ-5D scores were not significantly different at follow-up • PROMIS Function score significantly improved at follow-up compared to baseline																	
Pospiech et al. (59)	EEP	Q-TFA, EQ-5D-3L	Mean scores:					NR					• Patients with OI experienced fewer prosthesis-associated problems than socket prosthesis users. General quality of life, as assessed with the Problem score of the Q-TFA				• Patients with bone-anchored press-fit implants and patients with a socket-suspension system were group-matched for age, body mass index and mobility grade			
			Q-TFA																	
			Subscore		OI group	Socket group	Sig													
			PUS		89	87	n.s.													
			Mobility		87	79	*p* = 0.05													
			Problem		7	18	*p* < 0.001													
			Global		81	69	*p* = 0.022													
			EQ-5D-3L																	
			Subscore		OI group	Socket group	Sig													
			Index		0.89	0.93	n.s.													
			VAS		84.32	82.59	n.s.													
			• Patients with bone-anchored press-fit implants had a higher prosthesis-associated QoL when assessed with the Q-TFA • HRQoL as assessed with EQ-5D-3L was not different between groups																	
Örgel et al. (60)	EEP	Q-TFA, EQ-5D-5L, SAT-PRO^d^, PMQ^e^ 2.0, FIM^f^	Mean scores:					Mean score:					NR				• Greater mobility reported by the TOPS (OI) group and reduced problems score could possibly influence the higher level of satisfaction in this group			
			Q-TFA					PMQ 2.0												
			Subscore		OI group	Socket group	Sig			OI group	Socket group	Sig								
			PUS		NR	NR	n.s.	Score		NR	NR	*p* < 0.001								
			Mobility		NR	NR	n.s.	• Q-TFA mobility score showed a significant difference between the TOPS (OI) group and socket group; the TOPS group reporting greater mobility • Significantly higher PMQ 2.0 scores for the TOPS (OI) group than the socket group												
			Problem		NR	NR	*p* < 0.001													
			Global		NR	NR	*p* < 0.001													
			EQ-5D-5L																	
			Subscore		OI group	Socket group	Sig													
			Index		NR	NR	*p* < 0.004													
			VAS		NR	NR	*p* < 0.035													
			• Q-TFA problem score showed a significant difference between the TOPS (OI) group and socket group; the socket group reporting more problems • Q-TFA total score showed significant difference between the TOPS (OI) group and socket group; the TOPS group reporting better outcomes • EQ-5D-5L health and total scores showed a significant difference between the TOPS (OI) group and socket group. Higher scores were reported by the TOPS group																	
Welke et al. (61)	EEP	Q-TFA, SF-36, 6MWT, TUG	Mean scores:					Mean scores:					NR							
			Q-TFA					6MWT (in meters)												
			Subscore		OI group	Socket group	Sig			OI group	Socket group	Sig								
			PUS		85.0	89.2	n.s.	Distance		321.7	315.5	n.s.								
			Mobility		82.1	84.8	n.s.	TUG (in seconds)												
			Problem		10.3	17.3	n.s.			OI group	Socket group	Sig								
			Global		69.6	74.0	n.s.	Time		11.0	11.2	n.s.								
			SF-36					• No significant differences between groups reported between groups in 6MWT or TUG												
			Subscore		OI group	Socket group	Sig													
			PCS		46.3	46.9	n.s.													
			MCS		50.2	53.7	n.s.													
			PF		NR	NR	N/A													
			RP		NR	NR	N/A													
			BP		NR	NR	N/A													
			GH		NR	NR	N/A													
			VT		NR	NR	N/A													
			SF		NR	NR	N/A													
			RE		NR	NR	N/A													
			MH		NR	NR	N/A													
			• No significant differences between groups reported in SF-36 or Q-TFA scores																	
Atallah et al. (28)	OTN (reported as OFI-C for long femur and OFI-Y for short femur)	Q-TFA Prosthesis wearing time (PUS), health-related quality of life (GS)	Mean scores:					NR					• The Q-TFA mean prosthetic use score improved significantly at 1-year follow-up				• This study included those individuals who received modified ILP implants, i.e., curved osseointegration femur implant (OFI-C) indicated for a long femoral remnant and gamma osseointegration femur implant (OFI-Y) indicated for a short femoral remnant			
			Q-TFA																	
			OFI-C (*n* = 52)																	
			Subscore		Pre	Post	Sig													
			PUS		59	86	*p* < 0.01													
			GS		42	67	*P* < 0.01													
			OFI-Y (*n* = 16)																	
			Subscore		Pre	Post	Sig													
			PUS		31	93	*p* < 0.01													
			GS		31	79	*P* < 0.01													
			• The Q-TFA mean PUS and GS improved significantly at 1-year follow-up																	
Sinclair et al. (62)	POP	Q-TFA, 6MWT, Don/doff time	Mean scores:					Mean score:					NR				Mean score:			
			Q-TFA					6MWT (in meters)									Don/doff time (in seconds)			
			Subscore		Pre	Post	Sig			Pre	Post	Sig						Pre	Post	Sig
			PUS		78	96	n.s.	Distance		481	584	*p* < 0.001					Don	112.5	9.7	*p* < 0.05
			Mobility		64	81	n.s.										Doff	24.4	7.1	*p* < 0.05
			Problem		25	3	*p* < 0.001													
			Global		62	92	*p* < 0.001													
Davis-Wilson et al. (63)	Unspecified	WHODAS 2.0^g^, PLUS-M, ABC^h^	NR					Mean score:					NR				Mean scores:			
								PLUS-M									WHODAS 2.0			
										Pre	Post	Sig						Pre	Post	Sig
								T-score		48.67	58.60	*p* < 0.001					Score	11	5.33	*p* = 0.008
																	ABC			
																		Pre	Post	Sig
																	Score	72.78	88.67	*p* = 0.013

NR, Not reported.

^a^ 10 MWT, 10 m walk test.

^b^ LD-SRS, Limb Deformity–Scoliosis Research Society.

^c^ 2MWT, 2 min walk test.

^d^ SAT-PRO, satisfaction with prosthesis questionnaire.

^e^ PMQ 2.0, prosthesis mobility questionnaire 2.0.

^f^ FIM, functional independence measure.

^g^ WHODAS 2.0, World Health Organization Disability Assessment Schedule 2.0.

^h^ ABC, activity-specific balance scale.

### Health-related quality of life

Compared to baseline, an improvement in SF-36 physical component score (PCS) was reported at 1-year (54, 56), 2-year (44–47, 57), 5-year (46, 47), and 10-year (50) follow-ups. The SF-36 mental component score (MCS), however, was reported to have improved at a 2-year (57) follow-up in one study but did not consistently show improvements in other studies. The condition-specific HRQoL measure, Q-TFA global score, showed an improvement at 1-year (28, 51, 54–56, 58, 62), 2-year (44, 45), 5-year (46, 47, 52), 10-year (50), and 15-year (49) follow-up. Reduction in problems due to the prosthesis, measured by Q-TFA problem score, was reported at 1-year (48, 58, 62), 2-year (44, 45), 5-year (46, 47), 10-year (50), and 15-year (49) follow-up. Two articles (46, 47) presented 5-year follow-up data and reported the interim 2-year follow-up data. They reported that the differences between 2- and 5-year follow-ups for all SF-36 domains and Q-TFA subscales were not statistically significant. Similarly, there were no significant differences in these measures between 5- and 10-year follow-ups (50). A significant reduction in disability (as measured by the WHODAS 2.0) was also reported at 1-year follow-up (63).

Out of the four cohort studies, three reported on the differences in HRQoL outcomes between bone-anchored prosthesis and socket prosthesis users (59–61). Two studies (59, 60) reported that condition-specific HRQoL, as measured by the Q-TFA global score, was significantly higher, and problems related to prosthesis use were also significantly lower in the bone-anchored prosthesis cohort than those in the socket prosthesis cohort. One study (61) found no differences in these variables or the PCS and MCS in SF-36 or the Q-TFA global score. Out of the two cohort studies that also reported EQ-5D results (59, 60), one study (59) reported no significant difference in HRQoL between groups, whereas the other (60) showed a significant increase in the bone-anchored cohort than the socket cohort at 1-year follow-up. This increase could perhaps be due, in part, to a greater sample size (69 patients) in the latter study (60) than in the former (59), which had 39 patients, or because the individuals in the socket group in the former study (59) indicated that they were satisfied with their prostheses, whereas this was not controlled for in the latter study (60).

### Mobility and prosthesis use

Improvements in mobility were reported widely, as evident by the significant improvements in the distance walked during the 2MWT at 1-year follow-up (58) and 6MWT at 1-year (51, 54–56, 58, 62) and 2-year (57) follow-ups and improvements in TUG at 1-year follow-up (51, 55, 56). Studies on press-fit implants more commonly used performance-based outcome measures that specifically measured mobility and function. Although observer-based mobility performance measures were not used or reported by the studies on screw-type implants (44–50), information on mobility and prosthesis usage in these studies based on the self-reported Q-TFA did show improvements. Prosthesis use as measured by Q-TFA prosthetic use score was reported to have increased significantly at 1-year (28, 55, 58), 2-year (44, 45), 5-year (46, 47, 52), and 10-year (50), but not at 15-year (49), follow-up. Improved perceived mobility (measured by PLUS-M), balance (measured by ABC), and functional capacity were also reported at 1-year follow-up (63), as were significant reductions in time to don and doff the prosthesis (62).

Out of the four cohort studies, three reported on the differences in mobility between cohorts of bone-anchored prosthesis and socket prosthesis users (53, 60, 61). Gailey et al. (53) and Welke et al. (61) reported no significant differences in mobility between the two groups, as measured by the 10 MWT (53), 6 MWT (61), and TUG (53, 61) or self-perceived mobility, as measured by PLUS-M (53). Gailey et al. (53) reported on a small sample size (22 patients, 11 in each group) and did not report on the mean duration since the OI surgery for those in the bone-anchored prosthesis group. Additionally, the selection of participants is a limitation in Welke et al. (61). Individuals in the socket group in this study reported a high level of functional mobility and are not comparable to those socket users who face significant mobility issues due to their socket and may go on to benefit from bone-anchored prosthesis. Overall, cohort studies that report on comparisons of bone-anchored prosthesis users with socket users should be taken with caution, as individuals who are successful prosthesis users with a socket prosthesis are generally not considered candidates for bone-anchored prostheses.

Four out of seventeen single-arm trials (51, 55, 62, 63) and three out of the four cohort studies (53, 59, 61) reported on external prosthetic components. In the single-arm trials, three (55, 62, 63) reported that participants were fit with the same external components with the bone-anchored implant that they used with their pre-intervention socket system. One single-arm trial (51) did not clearly report this. In the three cohort studies that included details of external prosthetic components, the authors reported that the types of components were similar in both groups (OI and socket).

The evidence suggests that quality of life, mobility, prosthesis use, and satisfaction with the prosthesis improve with bone-anchored implants compared to the patients’ condition as socket prosthesis users. However, socket prosthesis users who do not face significant challenges with their sockets and already have a higher degree of mobility may not benefit as much, even if they opt for bone-anchored implants for prosthesis fixation.

### Complications and adverse events

Information on complications and their time frame can be useful in informing clinical decision-making, planning, and informing health economic models. Moreover, 17 out of the 21 articles on outcomes also reported on complications faced by patients. Table 4 summarizes the adverse events and complications reported in these articles. Nine case series and one cohort study reported only on infectious and other serious complications but presented no other outcomes of interest. Table 5 shows the findings of these studies and the odds of complications (where available) by implant type. The most commonly reported complication is superficial (skin/soft tissue) infections that occur in all types of implants from as few as 11% (44) to as much as two-thirds (52) of the patients. These complications are usually managed with oral or intravenous (parenteral) antibiotics or surgical intervention (such as debridement) (36). Soft-tissue refashioning (28, 52, 54, 56–58, 62, 63) and stoma hypergranulation (52, 55, 63) are other complications often reported. Mechanical complications, including the breakage of external parts, also occurred in third (58) to half (49) of the patients treated with BAP and were reported to be managed by exchanging percutaneous implant parts as needed. Such mechanical complications have been reported to increase between 5 and 10 years after implantation (50). More serious complications, such as implant loosening, implant breakage, and implant failure (requiring removal), were reported and were rarer in individuals treated with press-fit implants (ILP or OPL). Implant loosening has been reported to occur more frequently in screw-type implants and in the first 5 years following implantation. Implant removal occurred in 8 out of 51 patients in the OPRA (screw-type) study cohort (50) and as few as 2 out of 39 patients who were fitted with the ILP (press-fit type) implants (52). In the OPRA study cohort, half of the implant removals occurred in the first 5 years following implantation and the other half between 5 and 10 years (50).

**Table 4:** Adverse events and complications reported in included studies on clinical outcomes.

Study	Implant type	Uneventful course	Superficial/soft tissue infections	Deep infections	Periprosthetic fractures	Implant loosening	Implant breakage	Implant removal	Mechanical complications	Other
Screw-type fixation										
Hagberg et al. (44)	OPRA	NR	• In 2/18 patients	NR	NR	• In 1/18 patients	NR	NR	• 1/18 patients had a broken external component	NR
Brånemark et al. (45)	OPRA	NR	• 41 events in 28/51 patients (treated with oral antibiotics)	• In 4/51 patients (3 treated with antibiotics, 1 implant removal)	0	• In 3/51 patients leading to implant removal	NR	• In 4/51 patients (3 due to implant loosening, 1 due to deep infection)	• 4/51 patients experienced complications with the abutment and/or the abutment screw • Damaged components were replaced • No mechanical complications with the fixture	• 5 patients suffered episodic pain during rehabilitation, without loosening • 4 patients with 5 fractures; 3 in the ipsilateral hip, 1 below the elbow, and 1 vertebral compression
Brånemark et al. (46)	OPRA	NR	• 70 events in 34 patients (treated with oral antibiotics)	• 14 events in 11 patients (9 treated with oral antibiotics, 1 implant removal, 1 unresolved)	NR	• In 3/51 patients leading to implant removal	NR	• In 4/51 patients (3 due to implant loosening, 1 due to deep infection)	• 43 complications in 15 patients • Damaged abutment and/or the abutment screw were replaced • Incidences increased between 2- and 5-year post-OI	• Stump revisions in 3/51 patients • One of the deep infections caused early loosening/failure of the fixture
Matthews et al. (47)	OPRA	NR	• In 11/18 patients (treated with oral antibiotics)	• In 5/18 patients (2 patients treated with oral antibiotics, 3 requiring implant removal)	• 2 fractured the neck of the femur due to a fall	• In 1/18 patients (requiring removal)	NR	• In 5/18 patients (3 due to deep infections, 1 due to chronic pain, 1 due to implant fracture)	• 2 patients fell and fractured the abutment • 5 patients fractured the abutment-retaining bolt • 11 patients experienced abutment bending • Fractured components were replaced • 12 patients required surgery for abutment changes	• 1 of the superficial (soft-tissue) infection required operative debridement
Zaid et al. (48)	OPRA	NR	• 4 events in 4/9 patients (treated with oral antibiotics)	• In 1/9 patients (requiring implant removal)	• 1 intertrochanteric fracture close to the implant due to a fall	NR	NR	• In 1/9 patients due to deep infection	• 8/9 patients experienced problems with the connector, resulting in the replacement of the connectors	NR
Hagberg et al. (49)	OPRA	NR	NR	NR	NR	0	NR	NR	• Over 15 years, 61/111 patients had mechanical complications, resulting in the exchange of the percutaneous implant parts	NR
Hagberg et al. (50)	OPRA	NR	• Reported as 1.88 per 10 person-years	• In 16 patients (15 treated with antibiotics, 1 implant removal)	NR	• In 3 patients (in the first 5 years leading to removal)	• In 4 patients (requiring removal between 5 and 10 years)	• In 8 patients (3 due to implant loosening, 1 due to deep infection, 4 due to implant breakage)	• Mechanical complications of outer components (i.e., abutment or abutment screw) significantly increased (*p* = 0.001) in the 5–10-year period compared to the first 5 years after implantation	NR
Press-fit fixation										
Van de Meent et al. (51)	ILP	NR	• In 8/22 patients (managed by extensive cleaning with hydrogen peroxide and antibiotics as needed)	NR	NR	NR	NR	NR	NR	NR
Reetz et al. (52)	ILP	• In 9/39 patients	• 148 events in 26/39 patients (46 events did not require treatment, 85 events treated with oral, 7 with parenteral antibiotics, and 10 with surgical intervention)	• 8 events in 4/39 patients (1 did not require treatment, 5 events treated with oral antibiotics, and 2 with surgical intervention)	NR	• 1/39 patients experienced aseptic loosening within 1 year • 0/39 patients experienced septic loosening	•In 2/39 patients within 2 years (revised successfully)	•In 2/39 (due to pain)	• 12 dual-cone adapters broke in 9 patients and were replaced	• 30 events of soft-tissue refashioning in 14/39 patients due to stoma-redundant tissue. 1 patient experienced 9 events • 13 events of stoma hypergranulation in 8/39 patients
Gailey et al. (53)	ILP	No information on complications provided								
Al Muderis et al. (54)	ILP or OPL	• In 23/50 patients	• In 21/50 patients (13 treated with oral antibiotics, 5 to parenteral antibiotics, and 3 requiring debridement)	0	• In 4/50 patients due to falls (resolved successfully without removing the implant)	NR	NR	NR	NR	• Soft-tissue refashioning in 10/50 patients to avoid impingement, skin irritation, and infection • Revision of the implant was required in 2/50 patients; 1 due to failure of OI as a result of an undersized device, and 1 due to implant fatigue failure at 3.5 years
Leijendekkers et al. (55) *(information extracted from online supplemental materials accompanying the article)*	ILP or OPL	• In 19/31 patients (10 OPL, 9 ILP)	• 9/31 (2 OPL, 7 ILP) had low-grade soft-tissue infections treated with oral antibiotics • 1/31 (ILP) had high-grade soft-tissue infection requiring surgical intervention	NR	• In 4/31 (2 OPL, 2 ILP) patients due to falls	0	0	NR	• 2 dual-cone breakages with the ILP (none with the OPL), all successfully replaced	• Stoma hypergranulation in 2/31 patients (1 OPL, 1 ILP)
Al Muderis et al. (56)	OPL	NR	• 12 cases in 10/22 patients of low-grade soft-tissue infection • 3 cases in 2/22 patients of high-grade soft-tissue infection	0	0	0	0	0	NR	• 6/22 patients required refashioning surgery
McMenemy et al. (57)	OPL	NR	• Each patient had been prescribed a minimum of 1 course of empirical antibiotics for superficial infection	0	• In 1/7 patients (surgically stabilized with a dynamic hip screw which healed uneventfully)	0	NR	0	• 1/7 patients experienced a broken dual cone (after weightlifting), which was replaced	• 3/7 patients required refashioning of the stoma or stump 12–16 months postoperatively with no subsequent stump complications
Reif et al. (58)	OPL	NR	• 15 events in 9/18 patients. All treated with oral antibiotics	NR	• In 2/18 patients	0	0	0	• 6/18 patients experienced a broken attachment, which was replaced • 3/18 patients needed a longer dual cone	• 1/18 patients underwent stoma revision
Pospiech et al. (59)	EEP	No information on complications provided								
Örgel et al. (60)	EEP	No information on complications provided								
Welke et al. (61)	EEP	No information on complications provided								
Atallah et al. (28)	OTN (reported as OFI-C for long femur and OFI-Y for short femur)	NR	• In 13/68 patients (7 in OFI-C and 6 in OFI-Y)	NR	• In 2/68 patients (2 in OFI-C)	0	0	NR	• 3/68 patients experienced dual-cone adapter breakage	• Soft-tissue refashioning due to stoma-redundant tissue in 1/68 patients in the OFI-Y group • No individuals experienced multiple events of infections of the same grade
Sinclair et al. (62)	POP	NR	• 2 events in 1 patient	NR	• In 1/10 patients	• In 1/10 patients	NR	• In 2/10 patients (1 due to implant loosening, 1 due to periprosthetic fracture)	• 5 events in 2 patients (loose adaptors or outer adaptor bolts) which were replaced	• 2 patients underwent skin revisions to reduce redundant skin • 4 events of residual limb muscle pain, soreness in the residual limb, and anterior distal muscle pain
Davis-Wilson et al. (63)	Unspecified	NR	NR	NR	NR	NR	NR	NR	NR	• 1/9 patients experienced stoma hypergranulation and underwent soft tissue revision surgery • 1/9 patients experienced stoma pain • 1/9 patients experienced stoma erythema • 1/9 patients experienced stoma hematoma • 1/9 of patients experienced muscle spasms

NR, Not reported.

**Table 5:** Studies focusing specifically on complications and safety parameters.

Study	Implant type	Study design	OCEBM Level of Evidence	Aims/design	Number of patients	Findings	Odds of complications	Comments
Screw-type fixation								
Tillander et al. (36)	OPRA	Prospective case series	Level 4	• To explore infectious complications • The study group was followed prospectively for an average of 3 years to identify implant infections and cross-sectionally surveyed twice (at inclusion and after approximately 3 years) for bacterial presence, local infection, and antibiotic use	• 39 (33 with transfemoral level of amputation)	• 2/39 patients had infections at baseline and 7/39 patients had experienced infections at follow-up • 7 patients had a local infection at the skin penetration area in the 6-month period preceding baseline. Out of these 4 were treated with short-term oral antibiotics • 11 patients had a history of local infection at the skin penetration area during the 6-month period before follow-up. Out of these 6 patients were treated with short-term oral antibiotics • The most common bacteria found around the skin-implant interface were Staphylococcus aureus, coagulase negative staphylococci, and streptococci group A, B, or G	• This information was presented in another paper (67)	• These adverse events were not compared with those of conventional socket prostheses
Tillander et al. (67)	OPRA	Retrospective case series	Level 4	• To quantify the risk of osteomyelitis • To characterize the clinical effect of osteomyelitis (including risk of implant extraction and impairments to function) • To determine whether common patient factors (age, sex, body weight, diabetes, and implant component replacements) are associated with osteomyelitis in patients with transfemoral amputations	• 96	• Osteomyelitis occurred in 16/96 patients • Out of the 16, 10 patients underwent extraction of the fixture • Out of the remaining 6, prosthetic use was temporarily impaired in 4 patients with infection who did not undergo implant extraction	• The 10-year cumulative risk of implant-associated osteomyelitis was 20% (in 16/96 patients) • No significant association between osteomyelitis and age (being elderly), overweight (BMI >25 kg/m^2^), sex, or smoking	• None
Press-fit fixation								
Juhnke et al. (68)	ILP	Retrospective case series	Level 4	• To explored changes in clinical outcomes during the evolution of device designs and concurrent refinement of operative techniques: three systematic and empirically driven iterations over 15 years	• 69 • 30 in Group 1 (Design A or B) • 39 in Group 2 (Design C)	*Group 1:* • Only patients in this group needed reoperations or revisions due to infection • 1 structural failure of implant, 4 explanations, 3 fractures, 77% had intervention due to soft-tissue stoma and 80% due to “any unplanned intervention” *Group 2:* • 5 needed unplanned interventions. None of these surgeries were secondary to infections • No structural failures or explantations, 2 peri-implant fractures (did not require implant removal), and 1 intervention due to a soft-tissue stoma	NR	• Retrospective comparative analysis of patients treated over 14 years with three types of implant design • Implant design changes determined by clinical outcomes to reduce infection at the stoma and deep bone and implant interface
Al Muderis et al. (69)	ILP	Prospective case series	Level 4	• To report on the safety of press-fit osseointegrated implants used in Australia and the Netherlands	• 86 (65 males)	• 31/86 had an uneventful course with no complications • 29/86 developed low-grade or high-grade soft tissue infections that were managed with oral or parenteral antibiotics or surgical intervention (such as debridement) • 26/86 did not develop an infection but had one or more other complications requiring intervention, including stoma hypergranulation (17), soft-tissue redundancy (14), proximal femoral fracture (3), inadequate OI leading to implant replacement (1), implant breakage (2) • Mechanical complications: breakage of the pin used as a fail-safe mechanism (25) • 0/86 developed deep peri-implant infection • 1/86 underwent removal of the implant due to inadequate OI resulting from an undersized implant. This patient was re-treated with a larger-diameter implant	• Significant association between: ◦ Sex and risk of severe infection ◦ Females have a sixfold increase in risk • BMI or >25 kg/m^2^ and risk of mind infection. Threefold increase in risk of mild infections in these patients • Smoking and recurrent infections. Sevenfold increase in risk in these patients	• Participants were recruited in 2 centers (Australia and the Netherlands) and underwent the two-stage surgical procedure • This article developed and launched a classification system for infection based on clinical and radiographic signs to allow prospective incidence reporting, severity assessment • No significant association was observed between other characteristics and the risk of complications. Similar infection rates were observed at the two centers
Mohamed et al. (70)	ILP	Retrospective case series	Level 4	• To identify risk factors which lead to revision surgery after implant breakage • Rate and causes of revision surgery • Location of mechanical failure and septic loosening (intramedullary versus dual-cone adapter)	• 58 (41 males)	• 20/58 patients experienced implant failures • 7/20 had a failed intramedullary stem (6 due to breakages and 1 due to septic loosening). All 7 underwent revision surgery to have a larger-diameter OPL intramedullary stem because initial broken stem changed intramedullary space • 13/20 sustained dual-cone adapter breakages (9 due to weak-point breakage, 3 due to distal taper breakage, and 1 broke both). These were revised in an outpatient setting without the need for anesthesia • Factors associated with revision surgery identified as (1) stem failures due to smaller stem diameter, and (2) number of infectious events in a patient • Authors could not find evidence for any factor associated with dual-cone adapter weak-point or distal taper failure	NR	• Cold welding of the taper connection to the intramedullary stem was observed • After 9 years, the cumulative implant survival probability was 78% • Median implant survival time was 6 (IQR: 4) years
Hoellwarth et al. (71)	ILP or OPL	Retrospective case series	Level 4	• To assess the risk of periprosthetic fractures by a retrospective review identified 518 OI procedures which were undertaken in 458 patients between 2010 and 2018 for whom complete medical records were available • Potential risk factors including time since amputation, age at OI, bone density, weight, uni/bilateral implantation and sex were evaluated	• 313 with 347 femoral implants (279 unilateral, 34 bilateral) • 9 with 18 mixed transfemoral/transtibial implants	• No fractures occurred spontaneously • No patients required removal of implants • 22 periprosthetic fractures reported, representing 6.3% of 347 femoral implants • Fractures united in 21 out of 22 patients (95.5%) • 19/22 fractures due to ground-level fall, 2/22 due to twist, 1/22 due to kicking • The vast majority (19/22, 86.4%) occurred within 2 cm of the proximal tip of the implant and after a fall • Fixation most commonly involved dynamic hip screws (10) and reconstruction plates (9)	• Significant association between periprosthetic fractures and: ◦ Sex, a 3.89-fold increased risk of fracture for females ◦ Weight, a 1.02-fold increased risk of fracture per kg above a mean of 80.4 kg (*p* = 0.046) • No increased risk for bilateral implants time from amputation to OI, age at surgery, or bone density	• Mobility level and prosthesis wear time were negatively affected after fixation of the fracture in any patient • Given that the rate of fractures in lower-limb amputees using traditional socket prostheses has been reported to be 2% to 3%, OI consistently provides a better quality of life compared with traditional socket prostheses, and that even after a fracture mobility is likely to remain better compared with a traditional socket prosthesis
Wood et al. (72)	OPL	Prospective case series	Level 4	• To examine pain and pain management for up to 3 years after surgery in military persons with severe complex trauma-related (blast) injuries • Pain assessment using 4-point verbal rating scale (VRS) conducted (1) preoperatively, the day before surgery; and postoperatively at (2) discharge from hospital, (3) 6 weeks, (4) 3 months, and (5) 6 months	• 7 bilateral (all males)	• Progressive decrease in the use of regular analgesics • 6/7 patients reported no to mild pain on discharge from hospital to rehabilitation center (on average 17 days later) • 5/7 patients required some analgesic at 6 weeks postsurgery • 3/7 patients needed long-term pain management with opiates postoperatively. 2 were still using ay 3 months, and 1 continuing at 14 months postsurgery • 1/7 patients experienced persistent pain after discharge	NR	• Perioperative venous thrombosis or pulmonary embolism did not occur • 6/7 patients experiences systemic inflammatory response postoperatively • 1/7 patients experienced femoral shaft fracture during surgery which was managed conservatively • 3/7 patients had recurrent soft tissue complications. Two of these underwent revision surgeries within 18 months postsurgery • 3/7 patients experienced femoral fractures after discharge. 1 due to fall, 1 due to twist while standing, and 1 due to increased physical activity
Hoellwarth et al. (73)	OPL	Retrospective case series	Level 4	• To investigate the association between bone-anchored implants and mortality and assess the potential risk factors	• 485 (331 transfemoral, 154 transtibial)	• No deaths occurred intraoperatively or during inpatient recuperation or acute recovery after OI • 19 patients died after the OI procedure at a mean of 2.2 years after surgery (range: 58 days to 5 years) • 17 participants died of causes unrelated to OI • 2 died of direct OI-related infectious complications originating from the stoma site • Leading causes of death were cardiac issues (5/19), cancer (4/19), pulmonary issues (3/19), suicide (3/19), osseointegrated-related infection (2/19), and trauma (1/19)	• Factors that increase the risk: increased age (hazard ratio: 1.06), vascular disease (OR: 4.73), or amputation due to infectious causes (OR: 3.87) • Notable factors not associated with mortality risk included post-OI infection and sex	• Most patients who have had OI will most likely survive, but die of unrelated medical or accidental events • The incidence of suicide in this cohort highlights that those who have undergone amputation have long been recognized as a population at risk for mental health issues
Black et al. (74)	OPL	Retrospective case series	Level 4	• To assess the incidences, timelines, and risk factors of soft tissue complications in patients with lower limb prosthetic implants	• 60 (33 unilateral transfemoral, 2 bilateral transfemoral)	• Out of the 60 patients (combined data for transfemoral and transtibial levels): ◦ 25 developed soft tissue infections ◦ 5 developed osteomyelitis ◦ 6 had symptomatic neuromas ◦ 7 required soft tissue revisions • 47% of soft tissue infections occurred in the 1 month after implantation, and 76% occurred in the first 4 months	• Soft tissue infections were positively correlated with obesity (RR: 2.01) and female sex (RR: 2.15) • Neuroma development was associated with increased age at OI (RR: 1.09) • Osteomyelitis was positively correlated with decreased center experience (RR: 7.42)	• Patients were followed for an average of period of 21.8 months • Soft tissue infections were observed the soonest after implantation, with a median onset of 36 days after surgery • Deep infections of the bone and/or hardware occurred later in time, at a median of 157 days after surgery • Soft tissue redundancy and symptomatic neuromas appeared around 8 months and up to 18 months postoperatively • Hypertension, diabetes mellitus, tobacco use, and alcohol use did not have significant associations with poor outcomes • Increased center experience can reduce the risk of osteomyelitis
Örgel et al. (75)	EEP	Retrospective cohort study	Level 3	• To investigate the impact of periprosthetic fractures in persons who got an EEP between 2010 and 2017 at the 2 centers in Germany by comparing the outcomes in mobility [Prosthesis Mobility Questionnaire (PMQ), AMP K-level] and prosthesis wear time in hours in patients with a periprosthetic fracture to patients without a periprosthetic fracture • To derive a classification system and treatment algorithm of periprosthetic fractures related to TOPS	• 34 (15 in fracture group, 19 in control group)	• 15 periprosthetic fractures (5 intraoperative and 10 postoperative) • All postoperative fractures were treated with implant-retaining osteosynthesis • No implants required removal	• Sex- and age-related differences could not be evaluated as this study mainly consisted of younger (mean age: 48.7 years) and predominantly male (73.5%) patients	• For both the fracture and control group, a significant increase of the PMQ and K-level was observed before and after the OI treatment • Periprosthetic fractures do not worsen outcomes • A significantly higher increase of the PMQ before and after OI treatment for the group BMI ≥ 25 kg/m^2^ than for the group BMI < 25 kg/m^2^, regardless of a periprosthetic fracture • There was no significant improvement in the rehabilitation results for the K-level in favor of the overweight patients compared to the normal weight patients

NR, Not reported.

RR, Relative Risk.

OR, Odds Ratio.

Deeper infections affecting the residual femur are rare and appear more prevalent in screw-type implants, although it should be noted that the duration of follow-up is generally longer for these studies than for press-fit implants. The risk of osteomyelitis (deep infection of the bone) was studied in individuals with screw-type OPRA implants, and the 10-year cumulative risk was 20% (67). There was no significant association between osteomyelitis and advanced age, being overweight, patient's sex, or smoking. However, in a retrospective study of complications with the press-fit ILP implant, Al Muderis et al. (69) reported a threefold increase in the risk of mild infections in persons with overweight or obesity, a sixfold increase in the risk of severe infection in female patients, and a sevenfold increase in recurrent infections in patients who were smokers. The increased risk of soft-tissue infections in persons with obesity and in females who were treated with press-fit implants was also reported by another study (74).

Spontaneous periprosthetic fractures were reported to have not occurred with the OPL (71) in a retrospective case series of 347 patients. Periprosthetic fractures due to falls were rare and occurred in as low as 6.3% of patients treated with the OPL implant; however, the follow-up duration of these studies was short (1 or 2 years). The most common cause for a periprosthetic fracture is falling, and the most common location is close to the proximal tip of the implant (71). Females have approximately a fourfold greater increase in the risk of periprosthetic fractures; however, time from amputation to OI surgery, age at OI surgery, or bone density were not reported to be associated with increased risk for fractures (71). Periprosthetic fractures were reported to be managed successfully in most cases by uniting the bone with dynamic hip screws or reconstruction plates (71, 75) and have been reported to not worsen outcomes (75).

One study specifically examined pain and pain management for up to 3 years after OI surgery (72). This was conducted in a group of seven patients with military service experience who experienced severe complex trauma-related injuries and underwent bilateral transfemoral OI surgeries with the press-fit OPL implant. In this group, five patients were required to take analgesic at 6 weeks postsurgery, three needed long-term pain management with opiates (out of which only one continued opiate use at 14 months postsurgery), and one had persistent pain after discharge. A progressive decrease in the use of regular analgesics was reported.

The cumulative survival rate for the ILP implant after 9 years was reported to be 78% in a retrospective case series of 58 patients (70). In this group of 58 patients, approximately 35% experienced implant failures either due to intramedullary stem failure (12%) or mechanical complications, such as dual-cone adapter breakage (23%). Those who experienced stem failure underwent revision surgery to have a larger-diameter OPL stem implanted, and those who experienced mechanical complications were revised in an outpatient setting without anesthesia. It was determined that common factors that lead to implant/stem failure are the initial implantation of a smaller diameter stem or the number of infectious events in a patient. Improvements to the design of the implant have been credited to the reduction in unplanned interventions and structural failure requiring the removal of the implant (68).

A retrospective analysis of mortality in a cohort of 485 patients who received the OPL implant reported that no deaths occurred intraoperatively or during inpatient recuperation or recovery after OI surgery; however, 19 patients in this cohort died within 5 years after the OI surgery (73). Moreover, 17 out of these 19 died due to causes unrelated to OI surgery, and 2 died of infectious complications originating at the stoma site. One of these two deaths occurred between 2 and 5 years following the OI surgery and the other over 5 years after the surgery. Notably, among the seventeen patients in this cohort, three died of suicide, highlighting the need for mental health evaluation and support in persons with amputation. Although not included in the data extraction, there was a case report of a patient who died during the EEP surgical implantation due to a pulmonary embolism, which could have been related to a pre-existing risk of deep vein thrombosis and wheelchair immobilization. This case report recommended that additional preventive measures such as preoperative scoring systems and, in exceptional cases, using an inferior vena cava filter should be considered in patients with a high risk of developing venous thromboembolism.

### Patient experiences (qualitative literature)

Two research studies reported on the lived experiences of patients who received screw-type implants. Specific patient quotes from the qualitative studies to elucidate changes and challenges in the lives of the patients due to bone-anchored prostheses are included in Table 6. One study included three persons with upper-limb amputation (two transhumeral and one transradial) and ten persons with transfemoral amputations who used a bone-anchored prosthesis (76). The users described living with a bone-anchored prosthesis as a revolutionary change beyond functional improvements. Some users also described embodying the prosthetic leg as a part of them. The seven transfemoral BAP users in the other study (77) described the feeling of being *whole* again and described improvements in aspects of social participation, which greatly improved their quality of life. Both studies mentioned some challenges of being a bone-anchored prosthesis user, specifically due to the fear of infections, falling, and breaking the implant.

**Table 6:** Patient perspectives from qualitative research studies.

Study	Implant type	Patient quotes
Lundberg et al. (76)	OPRA	*Changes in life due to bone-anchored prostheses:* “I can feel that it’s (bone-anchored prosthesis) not as good as a healthy leg, but it’s far more normal than the old one (socket prosthesis). This is perhaps 70% as compared to a real leg and a real leg being 100% and an old prosthesis is perhaps 25%.” “The prosthesis (bone-anchored prosthesis) is a part of me since it works so well, and you don’t have to think that it’s a problem and that it should be hard and so forth … it’s more like a substitute, my “pretend leg”” “There is something missing, one part of me is missing and I miss it physically in a way I haven’t done before, not after the accident either. And this happened after I got the prosthesis (bone-anchored prosthesis) that is more me than ever, that makes me feel more whole as a person.” “I don’t think about having the prosthesis in that it doesn’t feel like a prosthesis. With this kind of technology you can’t feel it. I sit just as much on this leg as on the other leg and the scary thing was this week when I didn’t have my leg on, and when I suddenly stood up I felt I had on the prosthesis. It has come so far that the brain has also gradually begun to believe that I have a real leg” “…there is a fixture properly anchored, femur is reinforced with marrow and bone from the pelvis, it’s anchored with material from my own body, with the only purpose to give me the possibility to walk. It’s very concrete. As opposed to a traditional prosthesis that is slipped on to the outside of the body. But here I can feel when I put the foot down, so that I can feel the shock throughout the body, not in an unpleasant way but I feel it and it gives me a positive experience of my body as a whole.” “One part of the body is trapped in this vacuum-packed socket, that’s the way it’s. To be let out of this entrapment, just to feel the sun towards the thigh or the air that surrounds the thigh instead of this heat and the sweating that is coming. It was like … it was my definition of freedom, that and to not have to think about the suspension” “The other prosthesis ruled my life, it was my master in a way, it’s inevitable … it affected my mood and my interest in doing things that I knew would demand an extra effort. You had to weigh the pros and cons and that’s all gone now. Now it’s actually me … I am in command and not the left leg (S-prosthesis) and that’s a big difference.” *Challenges with bone-anchored prostheses:* “The disadvantage (with the OI-prosthesis) is that if you got stuck with the foot for instance which has happened a number of times, the leg is twitching (the fail-safe attachment device) and then you can’t turn it right unless you get to the prosthetic workshop and then you feel much more handicapped instead.’”
Hansen et al. (77)	OPRA	*Changes in life due to bone-anchored prostheses:* “I got sores from using the socket prosthesis, and I had major problems securing it because my limb is so short. Sometimes when I was working the prosthesis would just fall off. This one is easy to attach, and it does not fall off. Also, you don’t get any sores. Before the prosthesis was a barrier (socket-suspended prosthesis), now it’s a great help (osseointegrated prosthesis)” “The socket prosthesis cut me in the groin. It was very unpleasant, and I couldn’t do much. I couldn’t vacuum clean or mop the floors, it was impossible, I just couldn’t handle it. Today I can do all these things, and I don’t need help anymore.” “I used to do a lot of weight-lifting in the gym, and sometimes during the training my socket prosthesis would just fall off, and I just couldn’t live with that, because then I had to start all over again! When I have a prosthesis it has to work, and this new one does!” “And if we go for a walk, I’m able to hold my wife’s hand. I haven’t been able to do that for eight to ten years. Some people might think that isn’t a big deal, but to me it means a lot.” “This osseointegrated prosthesis has given me far more freedom and quality of life. I do not get chafes anymore, and I am not in pain. This means that I am able to do stuff with my kids again, and I am happier than before. Also, when I am together with my family and friends I am able to go for a walk after dinner instead of just staying at home reading a magasin.” “Well, I can’t deny the fact that I’m disabled. That’s obvious because I’m missing a leg. But with all the opportunities I’ve been given with this osseointegrated prosthesis, well, it almost makes up for my disability.” *Challenges with bone-anchored prostheses:* “I am an experienced dive instructor, but I am not able to go to the public pool, because there is an increased risk of infection if I jump into the water. That is a major disadvantage for me.” “I don’t go outside during winter as much as before after I got this prosthesis. If I fall my socket prosthesis would just fall off, but if I fall with this osseointegrated prosthesis there is a risk of breaking the implant. Therefore during winter I stay inside more, which of course is annoying.”

### Cost-effectiveness (health economic literature)

Five studies addressing the health economic impacts associated with bone-anchored implants for transfemoral prosthetic fixation were included. Table 7 outlines the study characteristics, main findings, and limitations. Two were cost-comparison studies (78, 82) and three were cost-utility studies (79, 81, 83). Two studies were based on the screw -type implant (78, 79), one on the OPL (81), and both did not specify the type of implant (82, 83). Another health economic evaluation was found but excluded as it combined the data for transfemoral and transtibial levels of BAP and separate data for transfemoral users was not available (84). To explore the cost-effectiveness of transfemoral BAP, it is essential that this information be analyzed separately.

**Table 7:** Health economic studies on bone-anchored implants for transfemoral prosthesis fixation.

Study (country)	Implant type	Aims	Funding	Patient population	Study design	Costs	Outcomes measured	Findings	Limitations and comments
Screw-type fixation									
Haggstrom et al. (78) (Sweden)	OPRA	To investigate the differences in prosthetic costs and service of osseointegrated prostheses compared to socket-suspended prostheses	Government and non-profit grants	50 patients with unilateral transfemoral amputation (36 socket-suspended prostheses, 20 osseointegrated prostheses, 6 patients used both kinds of prostheses)	Retrospective cost-analysis and survey on the number of visits	Retrospective costs over a 10- year period from one prosthetic workshop *Costs included*: New prostheses, services, adjustments, and repairs	Number of visits over a 10-year period from one prosthetic workshop	• 3.1 visits/year vs. 7.2 visits/year (cost-analysis), 3.4 visits/year vs. 9.2 visits/year (survey) • Mean total annual cost of new prostheses, services, repairs and adjustments was 14% lower for OI prostheses than socket prostheses (€3,149 and €3,672. respectively, n.s.) • Cost of material accounts for 92.5% for OI prostheses and 70% for socket prostheses	*Limitations*: • Did not include the costs of the bone-anchored implant, surgeries, hospital stay, postsurgical follow-up, rehabilitation, and the possible costs of dealing with complications
Hansson et al. (79) (Sweden)	OPRA	To compare the cost-effectiveness of treatment with a bone-anchored prosthesis and a socket-suspended prosthesis for patients with a transfemoral amputation	Government and non-profit grants	39 transfemoral prosthesis users followed for 2 years (80), no control group	Cost-utility analysis Perspective: Swedish healthcare system Time Horizon: 20 years Markov model based on clinical input from a 2-year follow-up study (80)	Costs acquired from hospital data, literature, and expert opinion *Costs included*: Socket: Acquired from (78), i.e., new prostheses, services, adjustments, and repairs Bone-anchored: Acquired from hospital data. OPRA implant, surgery, postsurgical care, medications, laboratory tests, and imaging	Health utility based on SF-6D were taken from a previous study by the same group (80)	• ICER (in 2009 euros): for bone-anchored prostheses was €83,374 per QALY gained compared with socket prostheses • Sensitivity analysis: the probability of bone-anchored prosthetic implants being cost-effective was reported to be 0.40 for a willingness-to-pay value of €48,000	*Limitations*: • Small sample size and they were only followed for 2 years, requiring extrapolation of the effects over time
Press-fit fixation									
Handford et al. (81) (UK)	OPL	To compare the cost of bone-anchored prostheses to the annual cost of a poorly-fitting socket	NR	80 transfemoral prosthesis users	Cost-utility based on retrospective analysis of costs and prospectively collected PROMs for health utility	Based on the UK Ministry of Defense fiscal costing. *Costs*: Bilateral Bone-anchored: £123,008 Unilateral Bone-anchored: £81,008 Socket: £9,624/year *Costs included*: Socket: Cost of multiple visits to the prosthetist, new molds, and prostheses Bone-anchored: Preoperative assessment, initial surgery, implant cost, hospital length of stay, two 4-week inpatient stays at a rehabilitation facility, seven follow-up appointments at a hospital, prosthetic follow-up, DEXA Scan, radiographs, and blood tests	Health Utility based on SF-36 scores converted to EQ-5D Health Utility Value	• Mean preoperative EQ-5D HUV was 0.64 which rose to 0.73 at 5 years and 0.78 at 6 years resulting in a cost/QALY of £96,129.71 and £40,040.92, respectively	*Limitations*: • To mitigate the impact of considerable variability in follow-up time points and the number of times each individual was followed up, the mean of all individuals EQ-5D-HUV was used for analysis. This could explain the failure to reach statistical significance in the pooled data, necessitating a subgroup analysis • Assumed that the ongoing high- cost of external components would be similar in socket and BAP users
Frossard et al. (82) (Australia)	Unspecified	Cost-comparison between socket and bone-anchored prostheses	NR	NR	Cost-comparison between socket and bone-anchored prostheses with three different types of knees	Historical costs based on administrative data from the Queensland Artificial Limb Service for provision of socket prostheses and simulated costs for bone-anchored prostheses over a 6-year cycle *Costs included*: Socket: liner, socket, knee unit, and foot unit Bone-anchored: Connector, knee unit, and foot unit	None	Bone-anchored prostheses were reported to be cost-saving by at least AUD 1,600 even with the most expensive knee, the microprocessor-controlled knee	*Limitations*: • Only ongoing costs associated with prostheses were considered and compared • Did not include the costs of the bone-anchored implant, surgeries, hospital stay, postsurgical follow-up, rehabilitation, and the possible costs of dealing with complications
Frossard et al. (83) (Australia)	Unspecified	To report the incremental costs, heath gain, and cost-effectiveness of BAP compared to socket-suspended prostheses	None	16 transfemoral prosthesis users	Cost-utility analysis Perspective: Queensland, Australia Time Horizon: 6 years	Costs based on administrative data from the Queensland Artificial Limb Service	Utility estimates from 2-year follow-up data from literature (44, 45) and multiplied over 6 years to obtain differences in QALYs	• ICER in 2016 AUD: AUD 16,632 per QALY gained • Bone-anchored prostheses were cost saving for 19% of patients and cost effective for 88% of patients	*Limitations*: • The analysis was based on a small convenient sample size and narrow case-mix • The cost data was based on a 6-year horizon for press-fit implants but the utility estimates were based on 2-year follow-up data with screw-type implants • Missing actual yearly costs were replaced with estimated costs based on their previous work (82) • Did not include the costs of the bone-anchored implant, surgeries, hospital stay, postsurgical follow-up, rehabilitation, and the possible costs of dealing with complications • No sensitivity analysis but presented ICERs based on scenarios varying the utility values

NR, Not reported.

Handford et al. (81) reported the cost of bilateral transfemoral OI to be £123,008 and unilateral to be £81,008. Haggstrom et al. (78) reported fewer visits to the prosthetist by those who use BAP vs. socket-suspended prostheses. Despite this, they reported that the costs of prosthetic materials and components were higher for BAP, which made the annual mean costs for bone-anchored and socket prostheses similar. The results of the three cost-utility studies vary greatly due to their methodological approaches. Hansson et al. (79) reported an ICER of €83,374 (in 2009 Euros) per QALY gained by bone-anchored prosthesis users over socket users. Frossard et al. (83) reported an AUD 16,632/QALY gained, and Handford et al. (81) reported £40,040.92/QALY 6 years after the OI surgery due to a steady increase in the patient-reported health utility value (HUV) in the 6 years postsurgery. The cost data in Frossard et al. (83) was based on a 6-year horizon for the press-fit implant, but the utility estimates were based on 2-year follow-up data with screw-type implant (44, 45) and multiplied over the 6 years to obtain differences in QALYs. Handford et al. (81) also presented the results of a subgroup analysis of patients grouped based on preoperative EQ-5D HUV being less or more than 0.60. The mean preoperative HUV of the group that had an HUV of <0.60 was 0.41, which reached 0.77 by 5 years and yielded a cost/QALY of £25,334.87. This, they reported, met the cost-effectiveness threshold of £30,000/QALY advised by NICE (85). The cost continued to fall in this group to neutrality with the comparator at 10.5 years. They concluded that those who perform poorly with socket prosthesis (typically those with an HUV of <0.60) are likely those who face significant challenges in walking or do not walk at all. Osseointegration offers the greatest benefit to these individuals as they continually show improvements in HUV and cost-effectiveness within 5 years. Conversely, in those with a preoperative HUV of >0.60, the gain in HUV and cost-effectiveness is less compelling.

Neither of the two cost-utility studies based on modeling (79, 83) included discounting of costs or outcomes. There is a notable range of prescribed rates for discounting costs and outcomes based on countries or regions (86–88), but these studies did not address discounting. As the failure of bone-anchored implants has been reported to be rare, the implants are estimated to maintain their effectiveness throughout the lifetime of an individual. Contrary to this, the majority of costs occur in the first year postsurgery. Due to this, the absence of discounting could potentially artificially inflate the reported ICER. Hansson et al. (79) presented alternative scenarios based on anticipated declines in utility in users of socket-suspension systems, as worsening of symptoms is expected and could cause a continuing decline in HRQoL in these users. This resulted in the cost per QALY gained of €37,020, €24,662, and €18,952, for a 1%, 2%, and 3% decline, respectively, over 20 years. Lastly, none of these studies take a societal perspective on costs and outcomes, which can be complex to acquire but provide a more holistic picture of the economic impact of bone-anchored prosthesis use.

## Discussion

Overall, bone-anchored implants that enable the direct attachment of prosthetic devices for individuals with transfemoral amputation who have failed conventional socket-suspension systems show promising results. The similarities in the patient selection criteria and the improved outcomes across the included studies add to the credibility of the findings on clinical efficacy. The evidence on clinical efficacy available on different implant types and on shorter (1- or 2-year) and longer (5-, 10-, and 15-year) follow-ups indicates that those who have been fitted with these implants consistently report improvements in quality of life, mobility, satisfaction with the prosthesis, and an overall improvement in situation as a person with an amputation. At 15-year postsurgery follow-up, approximately 64% of patients mentioned that osseointegration improved their overall situation as a person with amputation (49). It is noteworthy that studies that presented interim 2-year and 5-year follow-up data (46, 47) revealed no significant differences in HRQoL (measured by SF-36 domains and Q-TFA subscales) between these two time points. Similarly, there were no significant differences in these measures between 5- and 10-year follow-ups (50). These findings suggest that most advantages of bone-anchored prostheses can be expected within the first 2 years and are maintained beyond that. It is interesting to note that the mobility improvements may contribute to the concomitant improvements in patient-reported health-related quality of life. Mobility has been previously reported to be strongly positively correlated with general satisfaction and HRQoL in individuals with lower-limb prostheses (89). Improvements in mobility are further supported by other studies on BAP users who reported a higher daily step count and daily stepping time when assessing mobility in daily activities, i.e., not in a controlled lab setting (90). In addition, even those with bilateral transfemoral BAP after on average 7 years reported improved mobility (91).

It should be noted that although an improvement in quality of life has been reported, this often does not translate into an improvement in the mental health of BAP users. Only one study (57) reported an improvement in the mental component score of the SF-36, and none employed an instrument specifically designed to address changes in mental health. Mental health is a known challenge within the amputation and prosthesis-user communities (92–94). Depression has been reported to affect as many as one-third of persons with lower-limb amputation (92). More research is required to explore the mental health changes that accompany BAP use, as adequate evaluation and treatment of mental health concerns in this population may improve HRQoL.

Soft tissue infection is the most common complication consistently reported across studies, which is typically managed conservatively. The incidence of hypergranulation and the need for refashioning of the stoma or for soft tissue redundancy suggest that continuous efforts are required to improve and track soft tissue management. Of utmost concern is to continually track serious complications requiring implant removal. Survival rates of implants in the literature ranged from 78% to 99% for studies using press-fit implants (28, 52, 62, 70) and 72% to 92% for studies using screw-fit implants (45–47, 49). There seems to be an equal probability of implant loss for screw-type implants in the first 5 years and the subsequent 5 years in the one study that examined implant loss (50), so a longer-term tracking of these complications is crucial. Mechanical complications are common across implant types but were often reported to be managed by replacing external parts as needed. The incidences of mechanical complications increase between 5 and 10 years after implantation (50), and the cost of replacing external parts may lead to an increase in prosthetic care expenses over time. It has also been reported that at 5- and 10-year follow-ups, mechanical complications tend to be significantly correlated with prosthetic mobility or the occurrence of deep infections. Improved mobility that BAP offers to prosthesis users may therefore inadvertently contribute to mechanical complications (49). The statistics on mechanical complications of external parts need to be considered in the context of the expected longevity of any mechanical prosthesis component, which also needs periodic replacement in active socket prosthesis users.

Patient-reported experiences in the literature are based on screw-type implants and generally positive. In the future, additional qualitative studies on individuals who receive press-fit implants may be beneficial to enable the comparisons of patient perspectives and experiences. In addition, it would be beneficial to explore the changes and challenges that a patient experiences preoperatively and after receiving a BAP. The use of longitudinal qualitative research methods (95, 96) may be well positioned to understand the issues that socket prosthesis users experience and to articulate the changes that they experience when they transition to bone-anchored prosthesis.

The two cost-comparison studies (78, 82) have limited applicability for decision-making on increasing the availability of BAP. Cost-analysis or cost-comparison studies are considered appropriate when the outcomes of the intervention and the comparator are identical (97). It is evident from the information presented here that the outcomes of the socket-suspension and bone-anchored prostheses are not identical. The reports based on pre-/post-study designs illustrate that quality of life or mobility often changes when a previous socket-suspension system user becomes a bone-anchored prosthesis user. However, these two studies present useful information on some of the costs that are considered in the health economic evaluation of this technology.

Economic models/frameworks for evaluating costs and health outcomes differed across studies. The two cost-comparison studies (78, 82) and one of the cost-utility studies (83) did not include the costs of the bone-anchored implant, surgeries, hospital stay, postsurgical follow-up, and rehabilitation or the possible costs of dealing with complications. The results of these studies have limited usefulness and generalizability because of the narrow frame of costs (only prosthetic care costs) included for analysis. Without accounting for the upfront costs associated with bone-anchored implants (such as costs of the surgery, hospital stay, and postsurgical follow-up) and appropriate ongoing costs (such as those related to prosthetic care or dealing with complications), the results from these studies should be interpreted with caution. Not accounting for these costs likely led to an underestimation of the ICER. Hansson et al. (79) included these costs associated with bone-anchored implants, but they did not include the costs of many common complications, and their Markov model did not include many tunnel states in which patients often find themselves during their journey toward becoming BAP users. Handford et al. (81) included a broad list of costs in their analysis. The outcomes measured varied from the number of visits to a prosthetist (78) to utility values based on SF-6D (79) and EQ-5D (81).

Overall, the results from these health economic studies are mixed and complex to interpret. This necessitates future studies in this field to have health economics as a forethought and ideally be based on prospective real-world administrative data over a reasonable time horizon (at least 5 years). This may become increasingly feasible in the future with the growing adoption of electronic medical records. To acquire a more realistic picture of the cost-effectiveness of bone-anchored implants, the costs considered for analysis should include the cost of the implant, surgery, postsurgical care, rehabilitation, regular follow-up, and management of complications and should be compared against the costs borne by the system to service the needs of the socket-suspension system users, including their need for prosthetic services, medical follow-up, complication management, and surgical revisions. The HRQoL outcomes should be collected prospectively and should be generic to allow the calculation of utility values. The comparison of costs and outcomes should ideally be made with the patients’ pre-intervention state of socket prosthesis use, but in the absence of the availability of this information, to a control group matched on several parameters including similar functional mobility restrictions and similar types of prosthetic components. If modeling is deemed a more suitable tool to assist in decision-making, then it should account for many states to more accurately reflect the typical patient trajectory.

Nonetheless, overall, it appears that bone-anchored prostheses involve a higher upfront cost to the healthcare system but yield a longer-term gain, as evident by the improvements in health-related outcomes and reduced problems due to socket systems. Other groups, such as Ontario Health in Canada, concluded in their health technology assessment that bone-anchored implants are a cost-effective intervention (98). However, it is possible that this intervention is mostly cost-effective for those who stand to gain the most out of it, i.e., those who face significant challenges due to socket-suspension systems, and not suitable as primary treatment for prosthetic fixation.

The technology has evolved since the early 1990s with consistent revisions in design and improvements in outcomes for patients. Screw-type implants have longer follow-ups and have been around for a longer time, and the press-fit implants have higher reported case numbers and more comprehensive tracking of outcomes and complications. The press-fit implants have demonstrated a reduced risk of complications with concomitant improvements in quality of life and mobility. The latest iterations of both types of implants can also be used with individuals with a long or short length of the residual femur (28). The surgery can be done as a one-stage procedure that may reduce the burden on the patient and the healthcare system. A recent review presents evidence in favor of the one-stage approach owing to the lower incidence of postsurgical complications with this approach (99). Some work has been done to develop a comprehensive and systematic framework for tracking complications (69, 100) and a systematic outcomes-tracking framework (101, 102); however, not all centers follow the same guidelines for reporting. Future studies in this field can also further improve the quality of evidence by reporting on potential confounders (such as external prosthetic components, pre-existing pain, residual limb length, or bone mineral density) and addressing them by conducting and reporting subgroup analyses or other appropriate statistical tools, if statistical power allows.

As the number of cases increases across centers worldwide, there is also an opportunity to further explore changes in the mental health of prosthesis users and the factors/experiences contributing to changes in the perceptions of patients about their health-related quality of life. Well-designed mixed methods studies (103, 104) could address this need and contextualize the perceived changes in quality of life with patients’ experiences and challenges in their everyday lives. Future research on the lived experiences of patients and their caregivers and the impact of bone-anchored prostheses on productivity and vocational/employment situations will lead to a richer and more wholesome understanding of the change in the lives of patients that bone-anchored prostheses appear to promise.

The studies included in this review present considerable variability in follow-up duration, the type of variables on which data are collected, and the reported outcomes. The resultant inability to do a meta-analysis/synthesis may be perceived as a challenge for policymakers when deciding on the value of providing this technology; however, there is evidence that BAP seems to be a worthwhile alternative for those who are experiencing recurring issues with their socket prosthesis and can have a long-lasting impact on the individual's quality of life, function, and participation in society. As the body of evidence on clinical efficacy and complications evolves in this area, it would be prudent to adopt a standard suite of outcome measures and complication tracking at regular time points and for a longer term and to establish data reporting standards by consensus within the various centers around the globe offering this intervention. This will enable comparisons of outcomes across centers worldwide and across implant types. With such data in the future, a meta-analysis may also become feasible. When policymakers and regulatory bodies approve or implement this technology as a funded alternative intervention to socket prostheses for individuals experiencing recurring issues with their socket prostheses, it is essential that well-designed and planned cost-utility studies be conducted.

### Limitations

There were a few limitations of this review, primarily due to the types of study designs and reporting of information in the included studies. Despite similar measures being reported in studies with a pre-/post-design, a meta-analysis was not feasible due to the varying lengths of follow-up and the variability in how results were reported in the literature. Some articles only reported the statistical significance of the difference between the pre- and post-intervention but not actual values (47, 49, 56, 60), whereas others reported median scores and not mean scores (52, 55, 57). One of the issues that may impact the reported health-related quality of life was the persistence of phantom limb pain. This issue could not be fully explored in this review as this phenomenon is inconsistently reported in the included literature. Other potential confounders, such as residual limb length and type of prosthetic components, are insufficiently reported to allow accurate analysis of their potential impact on outcomes. It should be noted that this review excluded papers on gait parameters as this was recently reviewed (105) and the relationship between gait parameters and clinical outcomes needs to be further examined. Lastly, two of the included studies were reported to have been conducted as a clinical trial (48, 62), and one of these is under regulatory oversight by the FDA (62). These studies may be subject to different obligations to report outcomes and adverse events; however, we assessed their quality and risks of bias using appropriate tools.

## Conclusion

Overall, based on the information available presently, the clinical efficacy of bone-anchored prostheses is well established as hundreds of cases have been performed worldwide with beneficial outcomes for patients and complications being managed effectively. Patients also report positive changes in their lived experience. The evidence points to the cost-effectiveness of this technology for those who suffer poor outcomes with standard-of-care socket prostheses, although further work is needed to collect sufficient data for rigorous health economic analysis. Standardizing outcome tracking would help with synthesizing evidence across centers. This paper presents a single resource on data collected in this population that can be used for decision-making on the implementation of BAP for transfemoral amputation.

## Data Availability

The original contributions presented in the study are included in the article/Supplementary Material; further inquiries can be directed to the corresponding authors.
