# Supplementary material for: Bone-anchored prostheses for transfemoral amputation: a systematic review of outcomes, complications, patient experiences, and cost-effectiveness
Source: Front Rehabil Sci. 2024 Apr 2;5:1336042. doi: 10.3389/fresc.2024.1336042 (PMC11018971; doi:10.3389/fresc.2024.1336042)
Supplement: Supplementary file 1 [file Table1.pdf]

Supplementary Material

Bone-anchored prostheses for transfemoral amputation: A systematic review of outcomes, complications, patient experiences, and cost-effectiveness

Mayank Rehani*, Tania Stafinski, Jeff Round, C Allyson Jones, and Jacqueline S. Hebert*

*** Correspondence:** Jacqueline S. Hebert, MD FRCPC: jhebert@ualberta.ca
Mayank Rehani: rehani@ualberta.ca

Supplementary Table S1: Studies meeting secondary exclusion criteria

Citation	Reason for exclusion*
Hagberg K, Brånemark R. One hundred patients treated with osseointegrated transfemoral amputation prostheses-Rehabilitation perspective. *J Rehabil Res Dev*. (2009) 46(3):331–44. PMID: 19675986	Patients overlapped with and were included in the study by Brånemark et al., 2014 (45).
Hagberg K, Hansson E, Brånemark R. Outcome of Percutaneous Osseointegrated Prostheses for Patients With Unilateral Transfemoral Amputation at Two-Year Follow-Up. *Arch Phys Med Rehabil*. (2014) 95(11):2120–7. doi: 10.1016/j.apmr.2014.07.009	Patients overlapped with and were included in the study by Brånemark et al., 2014 (45).
Hagberg K. Bone-anchored prostheses in patients with traumatic bilateral transfemoral amputations: rehabilitation description and outcome in 12 cases treated with the OPRA implant system. *Disabil Rehabil Assist Technol*. (2019) 14(4):346–53. doi: 10.1080/17483107.2018.1449016	Patients overlapped with and were included in the study by Hagberg et al. 2008 (44) and Brånemark et al., 2014 (45).
Khemka A, Lord S, Bosley B, Al Muderis M. Osseointegrated prosthetic limb for amputees-over hundred cases. *Prosthet Orthot Int*. (2015) 39(Suppl 1):497. doi: 10.1177/0309364615591101	Conference abstract. Patients overlapped with and were included in the study by Al Muderis et al., 2017 (56).
Khemka A, Frossard L, Lord S, Bosley B, Al Muderis M. Health-related quality of life of individuals with transfemoral amputation fitted with the Transcutaneous Bone Anchoring Prosthesis following the OGAAP. *Prosthet Orthot Int*. (2015) 39(Suppl 1):465. doi: 10.1177/0309364615591101	Conference abstract. Patients overlapped with and were included in the study by Al Muderis et al., 2017 (56).
Al Muderis M, Lu W, Glatt V, Tetsworth K. Two-Stage Osseointegrated Reconstruction of Post-traumatic Unilateral Transfemoral Amputees. *Mil Med*. (2018) 183(Suppl 1):496–502. doi: 10.1093/milmed/usx185	Patients overlapped with and were included in the study by Al Muderis et al., 2016 (54). This article presented outcomes on a subset of 37 patients with traumatic etiology who were also included in Al Muderis et al., 2016. (54).

Citation	Reason for exclusion*
Gaffney BMM, Davis-Wilson HC, Awad ME, Tracy J, Melton DH, Lev G, et al. Daily steps and stepping cadence increase one-year following prosthesis osseointegration in people with lower-limb amputation. *Disabil Rehabil*. (2023):1–6. doi: 10.1080/09638288.2023.2200036	Patients overlapped with and were included in the study by Davis-Wilson et al., 2023 (63).
Black GG, Jung W, Wu X, Rozbruch SR, Otterburn DM. A Cost-Benefit Analysis of Osseointegrated Prostheses for Lower Limb Amputees in the US Health Care System. *Ann Plast Surg*. (2022) 88(3):S224–8. doi: 10.1097/SAP.0000000000003183	Presented combined results of cost-benefit analysis for transfemoral and transtibial levels but did not present results for transfemoral cases separately.
Örgel M, Ranker A, Harb A, Krettek C, Aschoff HH. Transkutane osseointegrierte Prothesensysteme (TOPS) nach Majoramputation der unteren Extremität. *Orthopade*. (2021) 50(1):4–13. doi: 10.1007/s00132-020-04031-2	Although English translation was acquired, this article presented complications and outcomes data for transfemoral, transtibial, and transhumeral (upper limb) levels but data for transfemoral cases was not separated.
Aschoff HH, Kennon RE, Keggi JM, Rubin LE. Transcutaneous, Distal Femoral, Intramedullary Attachment for Above-the-Knee Prostheses: An Endo-Exo Device. *J Bone Joint Surg Am*. (2010) 92(Suppl 2):180–6. doi: 10.2106/JBJS.J.00806	The study reported complication rates but was the interim report of another included study by Juhnke et al., 2015 (68) on complications.
Guirao L, Samitier CB, Costea M, Camos JM, Majo M, Pleguezuelos E. Improvement in walking abilities in transfemoral amputees with a distal weight bearing implant. *Prosthet Orthot Int*. 41(1):26–32. doi: 10.1177/0309364616633920	Intervention was not of interest. Study based on a bone-anchored implant aimed to enable distal weight bearing of the residuum within the socket, but not attach external prosthetic components.
Sullivan J, Uden M, Robinson KP, Sooriakumaran S. Rehabilitation of the trans-femoral amputee with an osseointegrated prosthesis: the United Kingdom experience. *Prosthet Orthot Int*. (2003) 27(2):114–20. doi: 10.1080/03093640308726667	Pre-post design suggested but no data presented with comparator.

Footnotes:

* Reference numbers in this column are based on those in the full article to minimize confusion due to renumbering.
