# Supplementary material for: Bone-anchored prostheses for transfemoral amputation: a systematic review of outcomes, complications, patient experiences, and cost-effectiveness
Source: Front Rehabil Sci. 2024 Apr 2;5:1336042. doi: 10.3389/fresc.2024.1336042 (PMC11018971; doi:10.3389/fresc.2024.1336042)
Supplement: Supplementary file 2 [file Table2.pdf]

Supplementary Material

Bone-anchored prostheses for transfemoral amputation: A systematic review of outcomes, complications, patient experiences, and cost-effectiveness

Mayank Rehani*, Tania Stafinski, Jeff Round, C Allyson Jones, and Jacqueline S. Hebert*

*** Correspondence:** Jacqueline S. Hebert, MD FRCPC: jhebert@ualberta.ca
Mayank Rehani: rehani@ualberta.ca

Supplementary Table S2: Risk of bias assessment and quality of literature on clinical outcomes

Study*	Implant type	OCEBM Level of Evidence	ROBINS-I Risk of bias due to…						
			Confounding bias	Selection bias	Information bias	Confounding	Selection bias	Information bias	Reporting bias
			Confounding[1]	selection of participants into the study[2]	classification of interventions[3]	deviations from intended intervention[4]	missing data[5]	measurement of outcomes[6]	selection of the reported result[7]
**Screw-type fixation**									
Hagberg et al. 2008 (44)	OPRA	Level 2	Moderate	Low	Low	Low	NI	Moderate	Moderate
Brånemark et al. 2014 (45)	OPRA	Level 2	Moderate	Low	Low	Low	Low	Moderate	Moderate
Brånemark et al. 2019 (46)	OPRA	Level 2	Moderate	Low	Low	Low	Low	Moderate	Moderate
Matthews et al. 2019 (47)	OPRA	Level 2	Moderate	Low	Low	Low	NI	Moderate	Moderate
Zaid et al. 2019 (48)	OPRA	Level 2	Moderate	Low	Low	Low	NI	Moderate	Moderate
Hagberg et al. 2020 (49)	OPRA	Level 2	Moderate	Low	Low	Low	Low	Moderate	Moderate
Hagberg et al. 2023 (50)	OPRA	Level 2	Moderate	Low	Low	Low	Low	Moderate	Moderate
**Press-fit type fixation**									
Van de Meent et al. 2013 (51)	ILP	Level 2	Moderate	Low	Low	Low	NI	Moderate	Moderate
Reetz et al. 2020 (52)	ILP	Level 2	Moderate	Low	Low	Low	Low	Moderate	Moderate
Gailey et al. 2023 (53)	ILP	Level 3	Low	Moderate	Low	Low	NI	Moderate	Moderate
Al Muderis et al. 2016 (54)	ILP or OPL	Level 3	Serious	Low	Low	Low	NI	Moderate	Serious
Leijendekkers et al. 2019 (55)	ILP or OPL	Level 2	Low	Low	Low	Low	Low	Moderate	Moderate

Study*	Implant type	OCEBM Level of Evidence	ROBINS-I<br>Risk of bias due to…						
			Confounding bias	Selection bias	Information bias	Confounding	Selection bias	Information bias	Reporting bias
			Confounding[1]	selection of participants into the study[2]	classification of interventions[3]	deviations from intended intervention[4]	missing data[5]	measurement of outcomes[6]	selection of the reported result[7]
Al Muderis et al. 2017 (56)	OPL	Level 3	Moderate	Low	Low	Low	NI	Moderate	Serious
McMenemy et al. 2020 (57)	OPL	Level 2	Moderate	Low	Low	Low	NI	Moderate	Moderate
Reif et al. 2021 (58)	OPL	Level 2	Moderate	Low	Low	Low	NI	Moderate	Moderate
Pospiech et al. 2021 (59)	EEP	Level 3	Low	Moderate	Low	Low	NI	Moderate	Moderate
Örgel et al. 2022 (60)	EEP	Level 3	Moderate	Moderate	Low	Low	Low	Moderate	Moderate
Welke et al. 2023 (61)	EEP	Level 3	Low	Moderate	Low	Low	NI	Moderate	Moderate
Atallah et al. 2020 (28)	OTN	Level 2	Moderate	Low	Low	Low	Low	Moderate	Moderate
Sinclair et al. 2022 (62)	POP	Level 2	Low	Low	Low	Low	Low	Moderate	Low
Davis-Wilson et al. 2023 (61)	Unspecified	Level 2	Low	Low	Low	Low	NI	Moderate	Moderate

Footnotes:

[*] Reference numbers in this column are based on those in the full article to minimize confusion due to renumbering.

[1] Generally, in single arm trials with pre-/post-comparison design where patients are serving as their own controls (in the pre-intervention stage), the impact of any baseline confounder can be considered minimal. In cohort studies, the risk of baseline confounder exists and subgroup analyses of potential confounders are an appropriate method of mitigating that risk. One of the potential confounders identified in this review was the reporting of external prosthetic components to which bone-anchored implants are attached. Switching to more sophisticated components (such as, microprocessor-controlled knees) post-osseointegration or a pre-existing difference between groups (in cohort studies) based on the type of prosthetic components was considered to have contributed to confounding. In this domain, a rating of 'Low' risk of confounding bias was assigned if confounding variables (such as type of external prosthetic components) were appropriately reported and considered to have made minimal impact to the external validity of the results. This would be the case if it was reported that all participants in a single arm trial had the same external prosthetic components pre- and post-osseointegration, or there was a statistical analysis demonstrating that the differences between groups on the type of

external components was not significant. A 'Moderate' risk of confounding bias was assigned if information about confounding variables was not reported, therefore having an unknown impact. This would be the case in studies where details about external prosthetic components were not at all reported or if the numbers of participants switching components post-intervention was not reported. A 'Serious' risk of confounding bias was ascertained when it had been reported that switching to superior prosthetic components post-OI occurred and may have had an impact on the reported findings.

[2] A rating of 'Low' was assigned if all participants who would have been eligible for the trial were included in the trial or if selection of the participants was reported to have occurred before the start of the intervention (which was the case in all included single arm trials), and if the start of follow-up and start of intervention coincides for all participants. A moderate risk of bias in this domain reflects that selection into the study may have been related to the intervention and outcome but appropriate measures were taken to adjust the selection bias.

[3] A rating of 'Low' was assigned in all included studies as the intervention status was well defined in all studies at the start of the study and intervention status could not have been affected by knowledge of the outcome.

[4] A rating of 'Low' was assigned for all included studies as any deviations from intended intervention reflected usual practice and the ability of the participants to adhere to routine follow-up was consistently a patient-selection criteria in the single arm trials.

[5] A rating of 'Low' was assigned if the proportions of and reasons for missing participants were similar across intervention groups (loss to follow-up/death) and 'NI' was assigned when no information is reported about missing data.

[6] A rating of 'Moderate' was assigned to the included studies because although the methods of outcome assessment were comparable across intervention groups (in single arm trials or cohort studies), many studies used PROMs or performance-based outcome measures reported by non-blinded clinicians, and the results of the outcome measure may have been influenced by knowledge of the intervention received by study participants.

[7] A rating of 'Low' was assigned if there was clear evidence (usually through examination of a pre-registered protocol or statistical analysis plan) that all reported results correspond to all intended outcomes, analyses and sub-cohorts. A rating of 'Moderate' in this domain was assigned when the outcome measurements and analyses are consistent with an a priori plan and there were no indications of selective reporting from multiple analyses or selection of cohorts/subgroups for analysis. A 'Serious' risk was assessed when outcomes were defined in different ways in the methods and results sections, or if there was a high risk perceived of selective reporting from among multiple analyses.
