# Supplementary material for: Bone-anchored prostheses for transfemoral amputation: a systematic review of outcomes, complications, patient experiences, and cost-effectiveness
Source: Front Rehabil Sci. 2024 Apr 2;5:1336042. doi: 10.3389/fresc.2024.1336042 (PMC11018971; doi:10.3389/fresc.2024.1336042)
Supplement: Supplementary file 3 [file Datasheet1.pdf]

Supplementary Material

Bone-anchored prostheses for transfemoral amputation: A systematic review of outcomes, complications, patient experiences, and cost-effectiveness

Mayank Rehani*, Tania Stafinski, Jeff Round, C Allyson Jones, and Jacqueline S. Hebert*

*** Correspondence:** Jacqueline S. Hebert, MD FRCPC: jhebert@ualberta.ca
Mayank Rehani: rehani@ualberta.ca

Supplementary Appendix S1: PRISMA Abstract Checklist

Topic	No.	Item	Reported?
**TITLE**			
**Title**	1	Identify the report as a systematic review.	Yes
**BACKGROUND**			
**Objectives**	2	Provide an explicit statement of the main objective(s) or question(s) the review addresses.	Yes
**METHODS**			
**Eligibility criteria**	3	Specify the inclusion and exclusion criteria for the review.	Yes
**Information sources**	4	Specify the information sources (e.g. databases, registers) used to identify studies and the date when each was last searched.	Yes
**Risk of bias**	5	Specify the methods used to assess risk of bias in the included studies.	Yes
**Synthesis of results**	6	Specify the methods used to present and synthesize results.	Yes
**RESULTS**			
**Included studies**	7	Give the total number of included studies and participants and summarise relevant characteristics of studies.	Yes
**Synthesis of results**	8	Present results for main outcomes, preferably indicating the number of included studies and participants for each. If meta-analysis was done, report the summary estimate and confidence/credible interval. If comparing groups, indicate the direction of the effect (i.e. which group is favoured).	Yes
**DISCUSSION**			
**Limitations of evidence**	9	Provide a brief summary of the limitations of the evidence included in the review (e.g. study risk of bias, inconsistency and imprecision).	Yes
**Interpretation**	10	Provide a general interpretation of the results and important implications.	Yes
**OTHER**			
**Funding**	11	Specify the primary source of funding for the review.	Yes
**Registration**	12	Provide the register name and registration number.	No
