# Supplementary material for: Bone-anchored prostheses for transfemoral amputation: a systematic review of outcomes, complications, patient experiences, and cost-effectiveness
Source: Front Rehabil Sci. 2024 Apr 2;5:1336042. doi: 10.3389/fresc.2024.1336042 (PMC11018971; doi:10.3389/fresc.2024.1336042)
Supplement: Supplementary file 5 [file Datasheet3.pdf]

Supplementary Material

Bone-anchored prostheses for transfemoral amputation: A systematic review of outcomes, complications, patient experiences, and cost-effectiveness

Mayank Rehani*, Tania Stafinski, Jeff Round, C Allyson Jones, and Jacqueline S. Hebert*

*** Correspondence:** Jacqueline S. Hebert, MD FRCPC: jhebert@ualberta.ca
Mayank Rehani: rehani@ualberta.ca

Supplementary Appendix S3: Literature search

Search number	Query	Search Details
18	#16 not #17	((("bone anchored prosthesis"[MeSH Terms] OR (("Prostheses and Implants"[MeSH Terms:noexp] OR "Artificial Limbs"[MeSH Terms] OR "Joint Prosthesis"[MeSH Terms] OR "Prosthesis Design"[MeSH Terms] OR ("prosthe*"[Text Word] OR "amput*"[Text Word] OR "artificial limb*"[Text Word]) OR (("limb"[Text Word] OR "limbs"[Text Word]) AND ("miss*"[Text Word] OR "loss"[Text Word] OR "lost"[Text Word] OR "lose"[Text Word]))) AND ("osseointegrat*"[Text Word] OR "osseo integrat*"[Text Word] OR "bone-anchored"[Text Word] OR "bone-anchored"[Text Word] OR "osseoanchor*"[Text Word] OR ("skelet*"[Text Word] AND ("fixed"[Text Word] OR "fixation"[Text Word]))))) AND ("bones of lower extremity"[MeSH Terms:noexp] OR "femur"[MeSH Terms] OR "foot bones"[MeSH Terms] OR "leg bones"[MeSH Terms] OR "lower extremity"[MeSH Terms] OR ("ankle"[MeSH Terms] OR "ankle joint"[MeSH Terms]) OR "foot"[MeSH Terms] OR ("knee"[MeSH Terms] OR "knee joint"[MeSH Terms]) OR "leg"[MeSH Terms] OR "thigh"[MeSH Terms] OR ("lower extremit*"[Text Word] OR "lower limb*"[Text Word] OR "ankle"[Text Word] OR "ankles"[Text Word] OR "foot"[Text Word] OR "feet"[Text Word] OR "knee"[Text Word] OR "knees"[Text Word] OR "leg"[Text Word] OR "legs"[Text Word] OR "thigh*"[Text Word] OR "femur"[Text Word] OR "tibia"[Text Word] OR "transfemora*"[Text Word] OR "trans femora*"[Text Word] OR "transtibia*"[Text Word] OR "trans tibia*"[Text Word]))) NOT ("animals"[MeSH Terms] NOT "humans"[MeSH Terms])) NOT ("child"[MeSH Terms] NOT ("adolescent"[MeSH Terms] OR "adult"[MeSH Terms]))
17	child[MeSH] not (adolescent[MeSH] or adult[MeSH])	"child"[MeSH Terms] NOT ("adolescent"[MeSH Terms] OR "adult"[MeSH Terms])
16	#14 not #15	(("bone anchored prosthesis"[MeSH Terms] OR (("Prostheses and Implants"[MeSH Terms:noexp] OR "Artificial Limbs"[MeSH Terms] OR "Joint Prosthesis"[MeSH Terms] OR "Prosthesis Design"[MeSH Terms] OR ("prosthe*"[Text Word] OR "amput*"[Text Word] OR "artificial limb*"[Text Word]) OR

		(("limb"[Text Word] OR "limbs"[Text Word]) AND ("miss*"[Text Word] OR "loss"[Text Word] OR "lost"[Text Word] OR "lose"[Text Word]))) AND ("osseointegrat*"[Text Word] OR "osseo integrat*"[Text Word] OR "bone-anchored"[Text Word] OR "bone-anchored"[Text Word] OR "osseoanchor*"[Text Word] OR ("skelet*"[Text Word] AND ("fixed"[Text Word] OR "fixation"[Text Word]))))) AND ("bones of lower extremity"[MeSH Terms:noexp] OR "femur"[MeSH Terms] OR "foot bones"[MeSH Terms] OR "leg bones"[MeSH Terms] OR "lower extremity"[MeSH Terms] OR ("ankle"[MeSH Terms] OR "ankle joint"[MeSH Terms]) OR "foot"[MeSH Terms] OR ("knee"[MeSH Terms] OR "knee joint"[MeSH Terms]) OR "leg"[MeSH Terms] OR "thigh"[MeSH Terms] OR ("lower extremit*"[Text Word] OR "lower limb*"[Text Word] OR "ankle"[Text Word] OR "ankles"[Text Word] OR "foot"[Text Word] OR "feet"[Text Word] OR "knee"[Text Word] OR "knees"[Text Word] OR "leg"[Text Word] OR "legs"[Text Word] OR "thigh*"[Text Word] OR "femur"[Text Word] OR "tibia"[Text Word] OR "transfemora*"[Text Word] OR "trans femora*"[Text Word] OR "transtibia*"[Text Word] OR "trans tibia*"[Text Word]))) NOT ("animals"[MeSH Terms] NOT "humans"[MeSH Terms])
15	animals[MeSH] not humans[MeSH]	"animals"[MeSH Terms] NOT "humans"[MeSH Terms]
14	#10 and #13	("bone anchored prosthesis"[MeSH Terms] OR (("Prostheses and Implants"[MeSH Terms:noexp] OR "Artificial Limbs"[MeSH Terms] OR "Joint Prosthesis"[MeSH Terms] OR "Prosthesis Design"[MeSH Terms] OR ("prosthe*"[Text Word] OR "amput*"[Text Word] OR "artificial limb*"[Text Word]) OR (("limb"[Text Word] OR "limbs"[Text Word]) AND ("miss*"[Text Word] OR "loss"[Text Word] OR "lost"[Text Word] OR "lose"[Text Word]))) AND ("osseointegrat*"[Text Word] OR "osseo integrat*"[Text Word] OR "bone-anchored"[Text Word] OR "bone-anchored"[Text Word] OR "osseoanchor*"[Text Word] OR ("skelet*"[Text Word] AND ("fixed"[Text Word] OR "fixation"[Text Word]))))) AND ("bones of lower extremity"[MeSH Terms:noexp] OR "femur"[MeSH Terms] OR "foot bones"[MeSH Terms] OR "leg bones"[MeSH Terms] OR "lower extremity"[MeSH Terms] OR ("ankle"[MeSH Terms] OR "ankle joint"[MeSH Terms]) OR "foot"[MeSH Terms] OR ("knee"[MeSH Terms] OR "knee joint"[MeSH Terms])

		OR "leg"[MeSH Terms] OR "thigh"[MeSH Terms] OR ("lower extremit*"[Text Word] OR "lower limb*"[Text Word] OR "ankle"[Text Word] OR "ankles"[Text Word] OR "foot"[Text Word] OR "feet"[Text Word] OR "knee"[Text Word] OR "knees"[Text Word] OR "leg"[Text Word] OR "legs"[Text Word] OR "thigh*"[Text Word] OR "femur"[Text Word] OR "tibia"[Text Word] OR "transfemora*"[Text Word] OR "trans femora*"[Text Word] OR "transtibia*"[Text Word] OR "trans tibia*"[Text Word]))
13	#11 or #12	"bones of lower extremity"[MeSH Terms:noexp] OR "femur"[MeSH Terms] OR "foot bones"[MeSH Terms] OR "leg bones"[MeSH Terms] OR "lower extremity"[MeSH Terms] OR "ankle"[MeSH Terms] OR "ankle joint"[MeSH Terms] OR "foot"[MeSH Terms] OR "knee"[MeSH Terms] OR "knee joint"[MeSH Terms] OR "leg"[MeSH Terms] OR "thigh"[MeSH Terms] OR "lower extremit*"[Text Word] OR "lower limb*"[Text Word] OR "ankle"[Text Word] OR "ankles"[Text Word] OR "foot"[Text Word] OR "feet"[Text Word] OR "knee"[Text Word] OR "knees"[Text Word] OR "leg"[Text Word] OR "legs"[Text Word] OR "thigh*"[Text Word] OR "femur"[Text Word] OR "tibia"[Text Word] OR "transfemora*"[Text Word] OR "trans femora*"[Text Word] OR "transtibia*"[Text Word] OR "trans tibia*"[Text Word]
12	"lower extremit*"[tw] or "lower limb*"[tw] or ankle[tw] or ankles[tw] or foot[tw] or feet[tw] or knee[tw] or knees[tw] or leg[tw] or legs[tw] or thigh*[tw] or femur[tw] or tibia[tw] or "transfemora*"[tw] or "trans-femora*"[tw] or "transtibia*"[tw] or "trans-tibia*"[tw]	"lower extremit*"[Text Word] OR "lower limb*"[Text Word] OR "ankle"[Text Word] OR "ankles"[Text Word] OR "foot"[Text Word] OR "feet"[Text Word] OR "knee"[Text Word] OR "knees"[Text Word] OR "leg"[Text Word] OR "legs"[Text Word] OR "thigh*"[Text Word] OR "femur"[Text Word] OR "tibia"[Text Word] OR "transfemora*"[Text Word] OR "trans femora*"[Text Word] OR "transtibia*"[Text Word] OR "trans tibia*"[Text Word]
11	"bones of lower extremity"[MeSH:noexp] or femur[MeSH] or "foot bones"[MeSH] or "leg bones"[MeSH] or Lower Extremity[MeSH] or Ankle[MeSH] or Foot[MeSH] or	"bones of lower extremity"[MeSH Terms:noexp] OR "femur"[MeSH Terms] OR "foot bones"[MeSH Terms] OR "leg bones"[MeSH Terms] OR "lower extremity"[MeSH Terms] OR "ankle"[MeSH Terms] OR "ankle joint"[MeSH Terms] OR "foot"[MeSH Terms] OR "knee"[MeSH Terms] OR "knee joint"[MeSH Terms] OR "leg"[MeSH Terms] OR "thigh"[MeSH Terms]

	Knee[MeSH] or Leg[MeSH] or Thigh[MeSH]	
10	#1 or #9	"bone anchored prosthesis"[MeSH Terms] OR (("Prostheses and Implants"[MeSH Terms:noexp] OR "Artificial Limbs"[MeSH Terms] OR "Joint Prosthesis"[MeSH Terms] OR "Prosthesis Design"[MeSH Terms] OR ("prosthe*"[Text Word] OR "amput*"[Text Word] OR "artificial limb*"[Text Word]) OR (("limb"[Text Word] OR "limbs"[Text Word]) AND ("miss*"[Text Word] OR "loss"[Text Word] OR "lost"[Text Word] OR "lose"[Text Word]))) AND ("osseointegrat*"[Text Word] OR "osseo integrat*"[Text Word] OR "bone-anchored"[Text Word] OR "bone-anchored"[Text Word] OR "osseoanchor*"[Text Word] OR ("skelet*"[Text Word] AND ("fixed"[Text Word] OR "fixation"[Text Word]))))
9	#7 and #8	("Prostheses and Implants"[MeSH Terms:noexp] OR "Artificial Limbs"[MeSH Terms] OR "Joint Prosthesis"[MeSH Terms] OR "Prosthesis Design"[MeSH Terms] OR ("prosthe*"[Text Word] OR "amput*"[Text Word] OR "artificial limb*"[Text Word]) OR (("limb"[Text Word] OR "limbs"[Text Word]) AND ("miss*"[Text Word] OR "loss"[Text Word] OR "lost"[Text Word] OR "lose"[Text Word]))) AND ("osseointegrat*"[Text Word] OR "osseo integrat*"[Text Word] OR "bone-anchored"[Text Word] OR "bone-anchored"[Text Word] OR "osseoanchor*"[Text Word] OR ("skelet*"[Text Word] AND ("fixed"[Text Word] OR "fixation"[Text Word])))
8	#5 or #6	"osseointegrat*"[Text Word] OR "osseo integrat*"[Text Word] OR "bone-anchored"[Text Word] OR "bone-anchored"[Text Word] OR "osseoanchor*"[Text Word] OR ("skelet*"[Text Word] AND ("fixed"[Text Word] OR "fixation"[Text Word]))
7	#2 or #3 or #4	"Prostheses and Implants"[MeSH Terms:noexp] OR "Artificial Limbs"[MeSH Terms] OR "Joint Prosthesis"[MeSH Terms] OR "Prosthesis Design"[MeSH Terms] OR ("prosthe*"[Text Word] OR "amput*"[Text Word] OR "artificial limb*"[Text Word]) OR (("limb"[Text Word] OR "limbs"[Text Word]) AND ("miss*"[Text Word] OR "loss"[Text Word] OR "lost"[Text Word] OR "lose"[Text Word]))

6	skelet*[tw] and (fixed[tw] or fixation[tw])	"skelet*"[Text Word] AND ("fixed"[Text Word] OR "fixation"[Text Word])
5	osseointegrat*[tw] or osseo-integrat*[tw] or "bone-anchored"[tw] or "bone anchored"[tw] or osseo-anchor*[tw] or osseoanchor*[tw]	"osseointegrat*"[Text Word] OR "osseo integrat*"[Text Word] OR "bone-anchored"[Text Word] OR "bone-anchored"[Text Word] OR "osseoanchor*"[Text Word]
4	(limb[tw] or limbs[tw]) and (miss*[tw] or loss[tw] or lost[tw] or lose[tw])	("limb"[Text Word] OR "limbs"[Text Word]) AND ("miss*"[Text Word] OR "loss"[Text Word] OR "lost"[Text Word] OR "lose"[Text Word])
3	prosthe*[tw] or amput*[tw] or artificial limb*[tw]	"prosthe*"[Text Word] OR "amput*"[Text Word] OR "artificial limb*"[Text Word]
2	"Prostheses and Implants"[MeSH:noexp] or "Artificial Limbs"[MeSH] or "Joint Prosthesis"[MeSH] or "Prosthesis Design"[MeSH]	"Prostheses and Implants"[MeSH Terms:noexp] OR "Artificial Limbs"[MeSH Terms] OR "Joint Prosthesis"[MeSH Terms] OR "Prosthesis Design"[MeSH Terms]
1	Bone-Anchored Prosthesis [MeSH]	"bone anchored prosthesis"[MeSH Terms]
